# Potential Mechanisms for Microbial Energy Acquisition in Oxic Deep-Sea Sediments

**DOI:** 10.1128/AEM.01023-16

**Published:** 2016-06-30

**Authors:** Benjamin J. Tully, John F. Heidelberg

**Affiliations:** a Center for Dark Energy Biosphere Investigations, University of Southern California, Los Angeles, California, USA; b Department of Biological Sciences, University of Southern California, Los Angeles, California, USA; University of Bayreuth

## Abstract

The South Pacific Gyre (SPG) possesses the lowest rates of sedimentation, surface chlorophyll concentration, and primary productivity in the global oceans. As a direct result, deep-sea sediments are thin and contain small amounts of labile organic carbon. It was recently shown that the entire SPG sediment column is oxygenated and may be representative of up to a third of the global marine environment. To understand the microbial processes that contribute to the removal of the labile organic matter at the water-sediment interface, a sediment sample was collected and subjected to metagenomic sequencing and analyses. Analysis of nine partially reconstructed environmental genomes, which represent approximately one-third of the microbial community, revealed that the members of the SPG surface sediment microbial community are phylogenetically distinct from surface/upper-ocean organisms. These genomes represent a wide distribution of novel organisms, including deep-branching Alphaproteobacteria, two novel organisms within the Proteobacteria, and new members of the Nitrospirae, Nitrospinae, and candidate phylum NC10. These genomes contain evidence for microbially mediated metal (iron/manganese) oxidation and carbon fixation linked to nitrification. Additionally, despite hypothesized energy limitation, members of the SPG microbial community had motility and chemotaxis genes and possessed mechanisms for the degradation of high-molecular-weight organic matter. This study contributes to our understanding of the metabolic potential of microorganisms in deep-sea oligotrophic sediments and their impact on local carbon geochemistry.

**IMPORTANCE** This research provides insight into the microbial metabolic potential of organisms inhabiting oxygenated deep-sea marine sediments. Current estimates suggest that these environments account for up to a third of the global marine sediment habitat. Nine novel deep-sea microbial genomes were reconstructed from a metagenomic data set and expand the limited number of environmental genomes from deep-sea sediment environments. This research provides phylogeny-linked insight into critical metabolisms, including carbon fixation associated with nitrification, which is assignable to members of the marine group 1 Thaumarchaeota, Nitrospinae, and Nitrospirae and neutrophilic metal (iron/manganese) oxidation assignable to a novel proteobacterium.

## INTRODUCTION

The South Pacific Gyre (SPG) covers approximately 10% of the Earth's surface and is the most oligotrophic marine environment in the surface oceans (1). Low-nutrient waters impact standing biomass and primary productivity, which in turn affect sedimentation rates and the amount of organic material exported to the deep ocean (2). Low sedimentation combined with water depths of >3,600 m directly result in shallow sediment coverage (3 to 130 m) of the basalt basement. The sediments within the SPG are oxygenated throughout the depth of the column into the basement environment, which is in sharp contrast to sediments beneath high-productivity oceanic regimes where oxygen is removed from the uppermost millimeters-centimeters (1, 3, 4). It has been estimated that up to a third of all deep-marine sedimentary environments are oxygenated, making them a globally relevant ecosystem (1).

**FIG 1:**
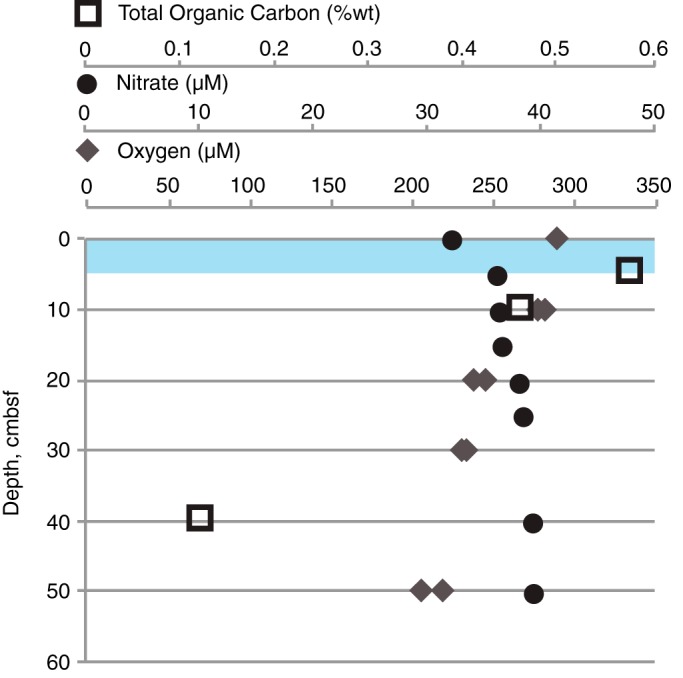
Depth profile of SPG site 10/U1369 with pore water measurements of nitrate, oxygen, and total organic carbon (TOC) based on published data (2). Overlying water measurements were used for zero cmbsf. No zero-cmbsf measurement for TOC was available. Sediment sample depth for DNA extraction is highlighted.

Microorganisms in SPG surface sediments are not limited by access to terminal electron acceptors (e.g., O_2_ and NO_3_^−^) (Fig. 1) or major nutrients (e.g., PO_4_^2−^; see Text S1 in the supplemental material) (1). However, standing biomass of microorganisms in surface sediments shows that the SPG contains approximately 2 to 4 orders of magnitude fewer cells per unit of volume than previously measured in marine surface sediment habitats (1, 2, 5), suggesting that the SPG sediments are depleted of sufficient organic energy inputs for a robust microbial population relative to high-productivity regions. The implication of these carbon-depleted conditions is that there is a direct impact on potential metabolisms and cellular functions of extant microorganisms in the surface sediment environment. It has been speculated that microbes under oligotrophic conditions will possess distinct genomic adaptations, such as the loss of motility mechanisms, decreases in lytic viral interactions, and a shift toward the utilization of recalcitrant organic compounds (6). Based on previous research, the SPG sediment column appears to be energy limited; measurements of total organic carbon (TOC) indicate that organic carbon is actively consumed for the first 20 cm below the seafloor (cmbsf), and it is at greater depths that TOC consumption diminishes to approximately zero (1, 2) (Fig. 1). It remains unclear whether the microbial community in the SPG surface sediments is composed of oligotrophic organisms, with limited metabolic potential, or copiotrophic organisms that possess a broad metabolic potential and can respond to sporadic organic matter inputs.

Previous research into the microbial communities using 16S small-subunit rRNA marker surveys (7–9) have shown that SPG surface sediments contain microorganisms belonging to the marine group 1 (MG-1) Thaumarchaeota, the Alphaproteobacteria, and the Gammaproteobacteria. While informative, these studies do not provide direct evidence of the metabolisms present. In an effort to capture the microbial metabolic potential linked with phylogeny and characterize how microorganisms influence the transformation of the available organic compounds within the sediment environment, a metagenomic sample was collected from SPG surface sediment to capture the metabolic potential of microorganisms without an *a priori* selection process. The metagenomic data set was assembled and binned to generate partial (<50% complete) and near-complete (≥90% complete) environmental microbial genomes and examined for metabolic capabilities related to carbon, nitrogen, and metal cycling. The metabolisms present in the reconstructed genomes from the SPG surface sediments have the potential for various organic matter transformations in deep-sea oxic sediment.

## MATERIALS AND METHODS

### Sample collection and geochemical data.

The sediment sample was collected as part of Expedition Knox-02RR to the SPG (December 2006 to January 2007 aboard the R/V Roger Revelle) from site 10 (39°18.617′ S, 139°48.036′ W) at 5,283 m depth, later designated U1369 during International Ocean Drilling Program (IODP) Expedition 329 (1, 10). The sediment was aseptically sampled from 0 to 5 cm from a multicore on the catwalk as the sample was brought onboard, using sterile, autoclaved cutoff syringes (10). Samples were stored at −80°C until DNA extraction. Pore water chemistry measurements were determined by the shipboard Scientific Party and are fully published (see Text S1 in the supplemental material) (10).

### DNA extraction and DNA amplification.

DNA was extracted from the 0- to 5-cm sediment samples using the PowerLyzer PowerSoil DNA kit (MO BIO Laboratories, Carlsbad, CA) by following the manufacturer's protocol. DNA then was quantified (3 μl) using the Qubit 1.0 fluorometer and the Qubit dsDNA HS assay kit (Life Technologies, Carlsbad, CA).

Amplification of extracted DNA was performed according to the Nugen Ovation ultralow library system protocol with slight modifications. In brief, 10 μl of template DNA (5.94 ng) was sheared to approximately 300 bp in size using the Covaris focused-ultrasonicator in 130-μl microTUBEs. Sonicator settings were 64 s at intensity 3, duty cycle of 5%, and bursts/cycle of 200. Further amplification steps strictly adhered to the Nugen prescribed protocol. It should be noted that all amplification steps have the potential to add unforeseen biases to environmental genomic studies, and while biases are known for linear amplification protocols, these biases remain unconstrained. To test for successful linear amplification of viable genomic DNA and exclude artifacts generated due to low input concentrations, amplified DNA was tested using 16S rRNA gene primers (7).

### MiSeq quality control and assembly.

Paired-end reads were generated from the sediment sample using 2-by-260 Illumina MiSeq library chemistry at the UC–Davis Genome Facility. Reads were trimmed off the Illumina adaptors using Cutadapt (v. 1.1; parameters: -O 12, -e 0.005) (11). Reads lacking the adapter were excluded from further analysis. The remaining reads were trimmed based on quality score using Btrim (v. 0.3.0; parameters: -w 20, -a 24, -S, -l 105) (12). Paired-end high-quality sequences were initially assembled into metagenomic contigs using IDBA-UD (v. 1.1.1; parameters: -maxk 250, -pre_correction, -min_pairs 0) (13), and all IDBA-UD-generated contigs were assembled using the Geneious *de novo* assembler (parameter: medium sensitivity/fast) (14).

### Composite genomes generated using ESOM.

Contigs were sized selected (>3 kbp), processed using the scripts provided as part of the tetramerFreqs/Binning package (https://github.com/tetramerFreqs/Binning) (15), and binned with Databionic emergent self-organizing map tools (ESOM; http://databionic-esom.sourceforge.net/) (16). Tetranucleotide frequencies were determined for the contigs by the esomWrapper.pl script. Information regarding the percent G+C (%G+C), percent amino acid usage (for the amino acids Lys, Arg, Thr, Glu, Asp, Ala, Val, and Ile [17]), and the depth of metagenomic sequence coverage, determined using BWA (v 0.6; aln parameter, -n 2; samse parameter, -n 2) (18), was added to the LRN file using the script addInfo2lrn.pl. The contig data were normalized using RobustZT and were trained using the k-batch algorithm (parameters: initial radius = 50, epochs = 20).

Putative coding DNA sequences (CDS) were determined for each contig using Prodigal (v 2.60) (19) and subsequently compared to the GenBank Reference Sequence (RefSeq) protein database (release 69) using BLASTP (parameters: -evalue 0.00005, -max_targets_seqs 5, -outfmt 5) (20). Based on the top five matches to the RefSeq database, MEGAN (v 4) (21) was used to determine the last common ancestor (LCA) for each putative CDS (parameters: recompute toppercent = 5, recompute minsupport = 1, collapse rank = species, select nodes = all). Using the hmm.majority.vote.pl script, part of the Multi-Metagenome package (https://github.com/MadsAlbertsen/multi-metagenome) (22), each contig was assigned a putative phylum-level taxonomic classification based on the most numerically abundant taxon of the putative CDS. These additional data were appended to the NAMES file. Phylogenetic bins then were determined manually using the ESOM map and taxonomic assignments as a guide.

Based on initial assessments, the number of contigs assigned as Proteobacteria dominated taxonomic assignments. The large abundance of Proteobacteria-assigned contigs increased the likelihood of incorrectly selecting boundaries around putative bins. In an effort to avoid cross-contamination, contigs that were assigned to “Other” taxonomic groups were used to construct a separate ESOM, which was constructed and assessed as described above. The Proteobacteria contigs were used to construct an ESOM, and a large group of unbinned *Alpha*- and Gammaproteobacteria contigs was identified. These class-assigned contigs were used to construct additional ESOMs, which were constructed and assessed as described above.

### Reliability of putative composite genomes.

Phylogenetic bins were initially assessed for how accurately a bin represents a putative composite genome using a protocol to determine genome completeness presented by Albertsen et al. (22). Briefly, putative CDS were determined for each contig using Prodigal and searched against a database of conserved marker genes using HMMER (v 3.1) (23). A first approximation of completeness was determined by comparing the number of identified genes in each bin to the total number of genes in the database. Small putative genomic bins with a limited number of identified conserved marker genes (<50% complete) were excluded from further consideration.

Further, an approximation of composite genome impurity was determined by identifying the number of genes from the database that were counted multiple times in a bin/genome. Additionally, genomic bins with a high degree of contamination (≥15 markers in duplication) were manually assessed to determine the merits of inclusion within this research based on the putative taxonomic assignments of marker and nonmarker CDS. Putative high-contamination bins determined to belong to a single taxonomic group were analyzed as a single unit. Individual contigs within a high-contamination group were assessed for taxonomic cohesiveness using a modified consensus voting technique from Albertsen et al. (22), as described above. Contigs that were not coherent with the overwhelming majority of the other contigs were removed from these unbinned taxonomic groups.

Putative genomic bins of sufficient size were analyzed using the lineage-specific workflow in CheckM to determine a more robust measurement of degree of completeness and contamination (24) (Table 1). Results from CheckM were confirmed using additional methodologies (data not shown; also see Text S1 in the supplemental material) (25).

**TABLE 1:** Statistics and phylogenetic assignments of analyzed putative genomes and bins

Genome/bin designation	Size (bp)	No. of putative CDS	No. of contigs	*N*_50_	Proposed phylogenetic placement	% complete^*a*^	% contamination^*a*^	% contamination attributed to strain heterogeneity^*a*^
SPGG1	2,851,473	2,709	299	11,329	Alphaproteobacteria	91.29	11.97	26.19
SPGG2	2,707,590	2,864	302	10,986	Novel proteobacterium	78.91	4.03	8.00
SPGG3	1,937,380	1,904	329	6,264	Nitrospina	70.62	5.13	0.00
SPGG4	1,446,879	1,485	288	5,023	Novel proteobacterium	64.71	4.23	10.53
SPGG5	1,333,276	1,381	257	5,439	Nitrospirae	61.31	1.41	0.00
SPGG6	1,255,507	1,289	126	12,965	Phylum NC10	61.31	0.85	0.00
SPGG7	1,800,395	1,673	259	7,654	Alphaproteobacteria	56.83	3.12	0.00
SPGG8	2,150,767	2,283	366	6,075	Novel bacterium	53.22	2.01	20.00
SPGG9	728,597	728	143	5,301	Chloroflexi	37.26	0.54	100.00
Unbinned Thaumarchaeota	4,974,130	6,214	642	8,816	NA	NA	NA	NA
Unbinned Gammaproteobacteria	8,288,222	8,415	1,415	6,136	NA	NA	NA	NA

a Calculations were determined by CheckM (24). NA, not applicable.

### Phylogenetic assignment.

**FIG 2:**
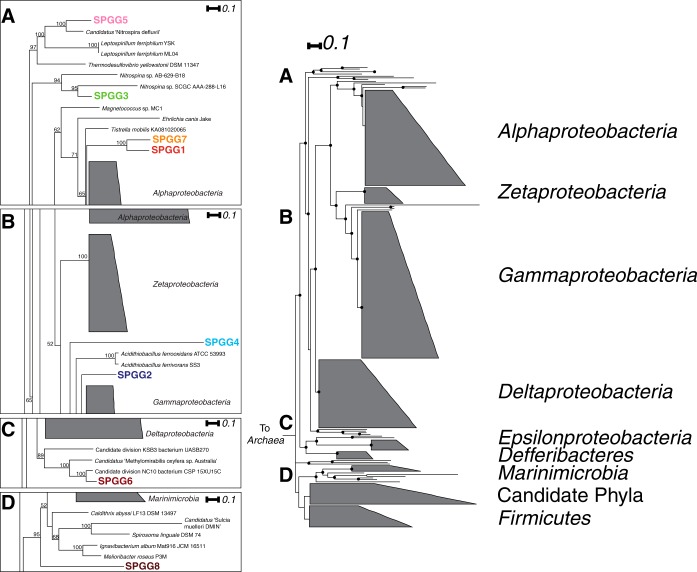
Phylogenetic tree constructed with RAxML based on eight concatenated phylogenetic marker (GyrB-RpoB-EFG-RpL4-RpL7-RpL11-RpS11-RpS13; 2,688-amino-acid alignment) sequences from a selection of 271 microbial genomes, including eight SPGGs (100 bootstrap replicates). Letters denote inset regions that were enlarged to highlight the immediate neighborhood of the SPGGs. Bootstrap values of ≥50 are indicated with black dots.

Putative CDS within each bin were examined for the presence of 10 different phylogenetic markers other than the 16S rRNA gene (26). The identified makers included DNA gyrase subunit B (GyrB), DNA-directed RNA polymerase subunit B (RpoB), transcription elongation factor G (EF-G), and five ribosomal proteins (RpL4, RpL7, and RpL11 and RpS11 and RpS13; see Table S1 and Data S1 in the supplemental material). These markers were identified in 271 bacterial genomes collected from the Integrated Microbial Genome (IMG) database (27). These 271 genomes were selected for their ability to reconstruct major bacterial groups from an initial set of >500 genomes that represented all of the major bacterial lineages. Bins with less than half of the phylogenetic markers were not included on the final tree. Phylogenetic markers were individually aligned using MUSCLE (28) and manually trimmed in Geneious (14). Phylogenetic markers were concatenated for a maximum alignment length of 2,688 amino acids, and maximum likelihood trees were generated using RAxML-HPC (v8.2.0) (29) and Nitrosopumilus maritimus SCM1 as an outgroup (PROTGAMMALG substitution model [30], 100 bootstrap replicates) (Fig. 2; see also Data S1). For bins with less than half of the eight phylogenetic markers, a separate tree was constructed using the same procedure, utilizing the markers EF-G, RpS11, and RpS13. Additionally, this tree was used to resolve phylogenetic placement for deeply branching, potentially novel clades (see Fig. S1). If present in a bin, the 16S rRNA gene was used to construct additional phylogenies. 16S rRNA gene sequences were compared to the SILVA (Ref123), GenBank RefSeq, and NCBI NT databases. Neighbors were selected using these methods and based on phylogenetic placement of the other identified marker genes. Sequences were aligned using MUSCLE (28). The alignments were trimmed manually to remove gaps in >50% of the sequences. 16S rRNA phylogenetic trees were constructed using PHYML (K80 substitution model; 100 bootstrap replicates) (see Fig. S2 and S3) (31).

### Relative abundance of microbial groups.

Putative CDS determined for assembled contigs using Prodigal were searched using HMMER (parameters: --cut_tc --notextw) against a set of 100 essential conserved markers described by Albertsen et al. (22). This marker set contains multiple ribosomal proteins and single-copy phylogenetic markers. Putative CDS identified as an essential conserved marker were assigned based on presence in a putative genome. Putative markers without an assignment to a genome were assigned a taxonomy based on a BLASTP search against the GenBank RefSeq database and the MEGAN4 LCA method (as described above). The high-quality metagenomic sequences were recruited against the identified putative marker CDSs using Bowtie2 (default parameters) (32), and the number of sequences recruited to each marker was determined. Length-normalized sequence counts for markers with the same taxonomic assignment at the phylum (or class for Proteobacteria) level were combined and relative abundance was determined.

### Functional annotation and genes of interest.

The RAST annotation service (33) was used to annotate the putative composite genomes (parameters: RAST annotation scheme = classic RAST, gene caller = RAST, FIGfam version = release70, automatically fix errors = Yes, backfill gaps = Yes, set verbose level = 0). Specific metabolisms of interest were assessed for each putative composite genome (see Table S2 in the supplemental material). Genes putatively involved in metal respiratory pathways, including the extracellular iron oxide respiratory system (MrtABC; MtoA), fungal laccases, fungal manganese peroxidases, and genes of the magnetosome operon, were identified in various reference genomes and aligned using CLUSTALW (34) to generate hidden Markov models (HMMs; hmmbuild; parameters: -amino, -informal afa) that could be searched against the putative CDS of each putative composite genome (hmmsearch; parameter, -E 0.00001; see Table S2). Further, BLASTP (-evalue 0.001) was used to identify genes that may represent environmentally relevant metabolisms partaking in carbon, nitrogen, and sulfur cycling (see Table S2). Positive matches were limited to matches with ≥30% amino acid identity (AAID) and ≥30% alignment length. Putative CDS were also processed using PSORTb (v.3) to determine predicted cellular localizations (35).

### Environmental MOBs involved in neutrophilic metal reactions.

Molybdopterin oxidoreductases (MOBs) identified in the Zetaproteobacteria member Mariprofundus ferrooxydans PV-1 (36) and homologs from five additional neutrophilic, iron-oxidizing microbial species were aligned, used to construct HMM models, and queried against the putative CDS of each putative composite genome (described above) (see Table S2 in the supplemental material). Additional reference MOBs with various prescribed functions were collected from GenBank. Reference sequences and environmental sequences were aligned using CLUSTALW (34) and manually trimmed. Environmental sequences with long branch lengths and/or poor placement among known MOB functions were removed, as were a cluster of environmental sequences putatively annotated as NADH-ubiquinone oxidoreductase chain G, in order to exclusively include metabolically relevant MOBs. A maximum likelihood phylogenetic tree was constructed using PHYML (31) (JTT substitution model, 100 bootstrap replicates) within Geneious (14) using the default settings.

### Accession number(s).

Raw reads (Sequence Read Archive [SRA] entry entries SRX1323577 and SRX1323576) and assembled contigs (Whole Genome Sequence [WGS] entry LKGT00000000) were submitted to NCBI under BioProject PRJNA297058. Assembled, unannotated sequences of the putative genomes were submitted to NCBI under BioProject entry PRJNA297058 and are associated with WGS accession numbers LXTD00000000 to LXTL00000000. RAST annotations, used for analysis, are available as GenBank files on FigShare (https://dx.doi.org/10.6084/m9.figshare.3219637.v1) and as an Excel file (see Data S2 in the supplemental material).

## RESULTS

### Sequencing, assembly, and binning.

A total of 62 million reads were generated from two MiSeq runs (16.3 Gbp). After trimming, a total of 46 million paired-end high-quality sequences (8.4 Gbp) remained. The initial assembly results from IDBA-UD generated 6,411 contigs >3 kbp in length (maximum, 40,691 bp; total, 29 Mbp; *N*_50_, 4,440 bp). After the second round of assembly utilizing the Geneious *de novo* assembler and all of the IDBA-UD contigs >500 bp in length (358,010 contigs), tetranucleotide frequency, amino acid usage, %G+C, and coverage were determined for 13,871 contigs >3 kbp in length (maximum, 70,476 bp; total, 77 Mbp; *N*_50_, 5,719 bp), which were then processed using an ESOM (see Text S1 in the supplemental material). Taxonomies were assigned to 98.0% of the contigs and used to guide binning.

Based on initial assessments of bin size and the number of conserved marker genes, 11 bins were included for further consideration. Two of the 11 bins were determined to have a high degree of contamination. Manual inspection revealed that multiple genomes from the same taxonomic group were present within the bins. Assemblies with incongruent taxonomic assignments were removed, and these bins were reclassified as “Unbinned Thaumarchaeota” and “Unbinned Gammaproteobacteria.” Based on a manual assessment of the conserved markers within each bin, it was determined that the unbinned Thaumarchaeota group contained at least three thaumarchaeotal genomes and the unbinned Gammaproteobacteria group contained at least two gammaprotoebacterial genomes.

The remaining nine bins were estimated to represent partial (<50% complete) to nearly complete (>90% complete) environmental genomes (Table 1). For seven of the bins, contamination estimates were low (<5%), while one bin had 5.13% contamination and another had 11.97% contamination. Some of the contamination in the bin with high contamination was likely the result of multiple, closely related genomes binning together, as strain heterogeneity accounted for an estimated 26.19% of the measured contamination (24). Based on these results, the putative bins were determined to represent high-quality environmental genomes and were given the designation South Pacific Gyre genome 1 through 9 (SPGG1-9).

### Phylogenetic assignments.

Based on the concatenated eight-phylogenetic-marker tree, eight of the nine SPGG were placed into seven phylogenetic groups (Fig. 2). SPGG1 and -7 branched together and were placed basal to most of the groups within the Alphaproteobacteria. SPGG2 and -4 constitute two deep branches within the Proteobacteria basal to the Gammaproteobacteria. SPGG2 and -4 had branch lengths similar to those of the class Acidothiobacillia, a distinct clade basal to the Gammaproteobacteria, suggesting that each genome represented a novel usbclass within the Proteobacteria. SPGG3 was determined to belong to the Nitrospinae and SPGG5 to the Nitrospirae. SPGG6 branched among members of the candidate phylum NC10. SPGG8 branched as a putative novel bacterium basal to the Marinimicrobia and a number of candidate organisms, including Caldithrix abyssi F13.

Due to the presence of an assembled 16S rRNA gene within the bin, SPGG6 (SPGG6_0506) could be more accurately assigned to group D within the candidate phylum NC10 (37). (Additional 16S rRNA gene comparisons were performed and phylogenetic trees constructed for SPGG1 [SPGG1_1410] and -3 [SPGG3_1465]; see Fig. S2 and S3 in the supplemental material.)

A second phylogenetic tree consisting of three concatenated phylogenetic markers was used to resolve the phylogeny of SPGG8 and assign phylogeny to SPGG9. With more specific/related references to resolve the phylogenies, SPGG8 branched basal to C. abyssi F13 and all members of the Latescibacteria and Marinimicrobia (see Fig. S1 in the supplemental material). SPGG9 deeply branched within the Chloroflexi, basal to the Dehalococcoides and several single-cell amplified genomes (see Fig. S1).

### Relative abundance of microbial groups.

**FIG 3:**
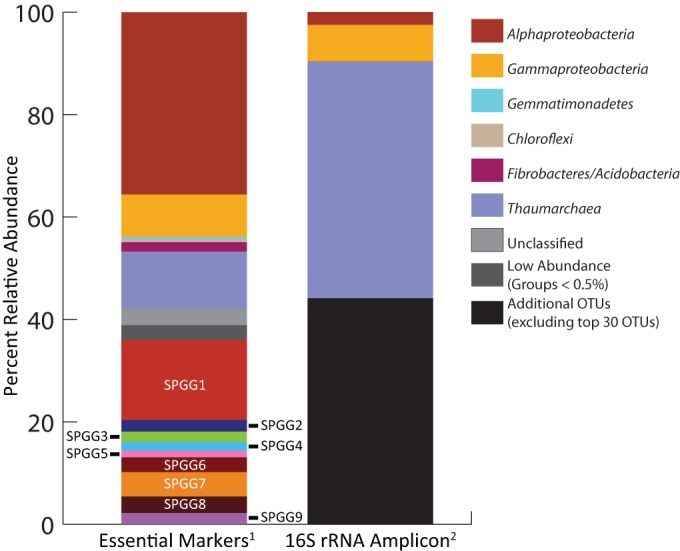
Bar graph representing the various recorded estimates of percent relative abundance of the SPG microbial population. Footnotes: 1, results based on the length-normalized recruitment of metagenomic sequences to conserved marker genes; 2, results taken from Tully and Heidelberg (7), combining the relative abundance of the taxonomic assignments of the 30 most abundant OTUs in the previous study.

From the Prodigal-derived putative CDS, based on a set of 100 essential markers, a total of 1,963 marker genes were identified. The SPGGs accounted for 684 of the markers (range, 54 to 107; mean, 76). The complete set of identified markers recruited 191,727 high-quality metagenomic sequence reads (0.42% of all sequences). The markers identified in the SPGGs accounted for more than a third (35.97%) of the length-normalized relative abundance (range, 1.28 to 15.61%; mean, 4.00%) (Fig. 3).

### Microbial metabolisms in South Pacific Gyre surface sediments.

The unbinned Thaumarchaeota possessed the potential for ammonia oxidation, with the presence of ammonia monooxygenase subunits ABC and the genes necessary for carbon fixation through the 3-hydroxypropionate/4-hydroxybutyrate (3-OH-prop/3-OH-buty) cycle. SPGG3 had a putative annotation for “Assimilatory Nitrate Reductase Large Subunit (EC 1.7.99.4)” (SPGG3_1079), and SPGG5 had a putative annotation for “Respiratory Nitrate Reductase Subunit, Conjectural (EC 1.7.99.4)” (SPGG5_0534). These two annotations represent different putative subunits of the nitrite oxidoreductase (NXR), the gene complex that has been identified to oxidize nitrite to nitrate (38, 39). The SPGG3 putative CDS has a top NCBI RefSeq match with 79% AAID to NxrA, the alpha subunit, within a Nitrospina sp. and clusters with Nitrospina NxrA sequences on a phylogenomic tree differentiating proteins related to nitrate reductases (NarG) and NxrA (see Fig. S4 in the supplemental material). The SPGG5 putative CDS is related to NxrC, the predicted gamma subunit of the NXR complex, and has a top match at 64% AAID to a hypothetical protein within Nitrospira moscoviensis and several putative NxrC sequences at similar AAID within other Nitrospira genomes, including “Candidatus Nitrospira nitrificans” (60% AAID) and “Candidatus Nitrospira nitrosa” (57% AAID). The role of NxrC has yet to be confirmed, but several homologs have been identified in each of the available Nitrospira genomes (40). Homologs for NxrA and NxrB were not identified in SPGG5. Additionally, SPGG5 contains ATP citrate lyase and 2-oxoglutarate oxidoreductase, genes necessary for carbon fixation via the reverse/reductive citric acid (rTCA) cycle. There is, however, no evidence of pyruvate synthase.

**FIG 4:**
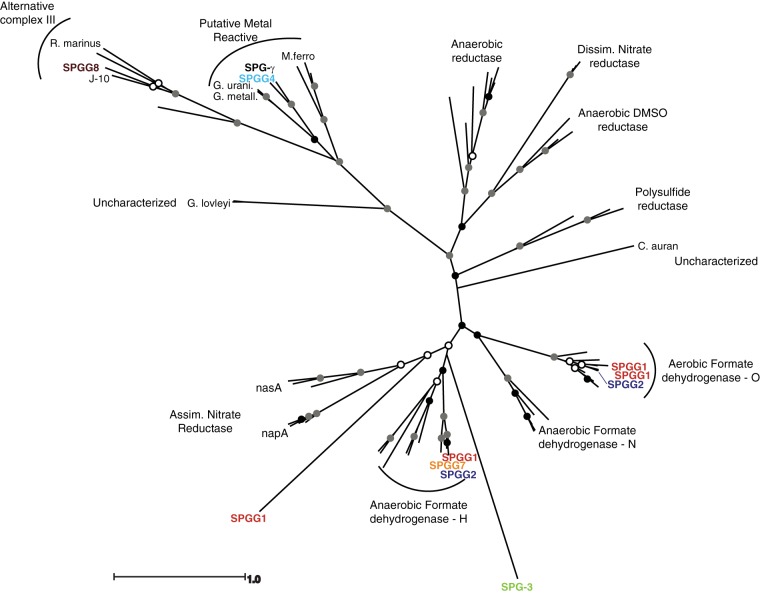
Maximum likelihood phylogenetic tree generated using PHYML based on molybdopterin oxidoreductase sequence (685-amino-acid alignment) for 67 proteins, including 10 putative CDS from the SPGGs (100 bootstrap replicates). Black circles, 100% branch support; gray circles, ≥75% branch support; white circles, ≥50% branch support. Abbreviations: SPGG-γ, unbinned Gammaproteobacteria; C. auran., Chloroflexus aurantiacus J-10; R. marinus, Rhodothermus marinus; G. urani., Geobacter uraniireducens; G. metall., Geobacter metallireducens; M. ferro., Mariprofundus ferrooxydans PV-1; *napA*, nitrate reductase; *nasA*, nitrate reductase; DMSO, dimethyl sulfoxide; Assim., assimilatory; Dissim., dissimilatory.

SPGG4, a previously unidentified proteobacterium, contained a MOB that branches with orthologs that have been identified as part of the electron transport chain in some bacteria capable of neutrophilic Fe(II) oxidation (Fig. 4, SPGG4_0366) (41). However, SPGG4 does not possess homologs to the c-type cytochromes identified as an essential element of the electron transport chain within Mariprofundus ferrooxydans PV-1 (41). SPGG4 did possess a diheme-containing, annotated c_4_-type cytochrome (SPGG4_1107), but this putative gene did not have significant AAID (<20%) to the M. ferrooxydans PV-1 genes. An additional homolog of this putative Fe(II) oxidation-related MOB was also present among the unbinned Gammaproteobacteria, although no M. ferrooxydans PV-1-related cytochromes were identified. SPGG4 has the potential to convert methanol to formaldehyde to formate via annotated methanol dehydrogenase (SPGG4_0512) and quino(hemo)protein alcohol dehydrogenase (SPGG4_1365). Based on the genome sequence, SPGG4 possessed a formate dehydrogenase (EC 1.2.1.2; SPGG4_0803) which will consume formate, generating H^+^ and CO_2_. SPGG4 possessed formylmethanofuran-tetrahydromethanopterin (FMR-THMPT) formyltransferase (SPGG4_1244) and formylmethanofuran dehydrogenase (SPGG4_1243), which can reversibly convert FMR-THMPT to FMR to formate. SPGG4 had an annotated Ni-Fe hydrogenase (SPGG4_1032-1036) which can reversibly oxidize molecular H_2_ to produce H^+^ and H_2_O.

### South Pacific Gyre genomes: evidence of energy limitation?

SPGGs were assessed for genomic signatures that may be indicative of energy limitation, including motility, viral interactions (see Text S1 in the supplemental material), and organic compounds utilized for microbial metabolism. Based on the annotations, five of the SPGGs had putative flagellum biosynthesis genes and/or genes involved with gliding motility (Table 2). An SPGG genome was considered to possess the genes for flagellum biosynthesis only if it possessed a subset of genes that were part of the *fli*, *flg*, *flh*, and/or *mot* operons. Gliding motility was determined based on the combined presence of *gldGF* and genes annotated as “gliding motility-related protein” or *sprT*. Four of the SPGGs had genes related to chemotaxis, determined by the presence of genes in the Che pathway, TonB-dependent receptors, cyclic AMP receptor proteins, and/or methyl-accepting chemotaxis proteins.

**TABLE 2:** Occurrence of genes of interest for motility, chemotaxis, extracellular protein interactions, and oligosaccharide degradation for the putative SPG genomes

Category	PFAM	Presence/absence (total) or % (total)								
		SPGG1	SPGG2	SPGG3	SPGG4	SPGG5	SPGG6	SPGG7	SPGG8	SPGG9
Motility and chemotaxis										
Flagellum		+	−	+	−	+	−	−	+	−
Gliding motility		−	−	−	+	−	−	−	+	−
Chemotactic systems		+	−	+	−	+	−	−	+	−
Extracellular protein interactions										
Outer membrane-bound proteases (total, if >1)		+ (2)	+ (2)	+	+	+	+	−	−	−
Dipeptide transporter(s)		+	+	+	+	−	+	+	−	+
Oligopeptide transporter(s)		+	+	+	+	−	+	+	+	+
% ABC-type related to transport (total no.)^*a*^		43 (92)	44 (79)	44 (27)	37 (26)	22 (23)	24 (29)	32 (41)	31 (36)	72 (25)
Oligosaccharide-degrading enzymes										
CAZy family name and general function										
GH1, beta-glucosidase	PF00232.12	−	−	−	−	−	−	−	−	+
GH2, beta-galactosidase	PF00703.15	−	−	−	−	−	−	−	+	−
GH3, beta-hexosaminidase and beta-*n*-acetyl-glucosaminidase	PF00933.15	−	+	−	−	+	−	+	−	−
GH39, alpha-l-iduronidase and beta-xylosidase	PF01229.11	−	+	−	−	−	−	−	−	−
Phenolic compound degradation domains										
Laccase	PF02578.9	+	+	+	+	+	+	−	−	−
Dioxygenase	PF00775.15	−	+ (2)	−	−	−	−	−	−	−
Peroxidase	PF00141.19	+ (2)	−	−	−	−	−	+	−	−

a Percent ABC-type transporters related to amino acid and peptide transport (total number of identified ABC-type subunits).

SPGG annotations were searched for exoproteases and ATP-binding cassette-type (ABC-type) transporters predicted to mobilize amino acids and/or peptides across cell membranes. Six of the SPGGs had at least one annotated peptidase with predicted localization attached to the extracellular membrane (Table 2). With the exception of SPGG5 (Nitrospirae), all SPGGs had annotated oligopeptide and/or dipeptide ABC-type transporters. Further, an assessment of all annotated ABC-type transporter components (permease, substrate binding, and ATP binding) revealed that 22 to 72% of SPGG ABC-type components were associated with amino acid and/or peptide transport (Table 2; also see Table S3 in the supplemental material).

The SPGGs were also searched for genes putatively related to the degradation of recalcitrant carbon, including those previously identified in coastal sediments (42), such as cellulose, pectin, chitin, phenolic compounds, and oligosaccharides. The recovered segments of the SPGGs did not contain any putative genes associated with cellulose, hemicellulose, or pectin degradation. The unbinned Thaumarchaeota possessed an annotated chitinase, and SPGGs from the Proteobacteria, Nitrospirae, Chloroflexi (SPGG2, -7, -5, and -9, respectively), and a novel bacterium (SPGG8) had 1 to 2 domains within putative CDS capable of oligosaccharide degradation (Table 2) (42). All but SPGG8 and -9 had functional domains identified as laccases, dioxygenases, and/or peroxidases (Table 2).

The SPGGs were also searched for genes related to known anaerobic metabolisms, including denitrification, sulfate reduction, Fe(III) reduction, and methanogenesis (see Table S2 in the supplemental material). There were no matches for genes related to sulfate or Fe(III) reduction. There was no other indication of methanogenesis except for the previously mentioned FMR dehydrogenase and FRM-THMPT formyltransferase. Both the unbinned Thaumarchaeota and SPGG7, an unclassified alphaproteobacterium, possessed putative CDS that matched nitrite reductase (NirK; SPGG7_1090), while the unbinned Gammaproteobacteria contained a putative CDS that matched nitric oxide reductase (NorB). Both unclassified Alphaproteobacteria (SPGG1_1859 and SPGG7_0816) and one of the novel proteobacteria (SPGG2_0455) possessed MOBs that cluster with known anaerobic formate dehydrogenases (FDH-H). SPGG1 (SPGG1_0055 and SPGG1_2670) and SPGG2 (SPGG2_0083) possessed MOBs that cluster with known aerobic formate dehydrogenases (FDH-O).

## DISCUSSION

Nine putative microbial genomes, representative of approximately 36% of the total microbial abundance, were identified from a metagenomic data set sampled from the oxic SPG surface sediments. With the exception of SPGG8, all other SPGGs could be placed within known, defined phylogenetic groups (Fig. 2; also see Fig. S1 to S3 in the supplemental material). SPGG8 was related to the Marinimicrobia and Caldithrix as well as a number of other candidate phyla; however, the placement of SPGG8 suggested that it represents a novel organism. This is supported by the second phylogenetic tree, constructed using EF-G, RpS11, and RpS13, that places SPGG8 basal to C. abyssi F13 and the Latescibacteria (see Fig. S2). SPGG6 clustered among members of the candidate phylum NC10. To date, only three other NC10 genomes are available (references 37 and 43 and unpublished [IMG taxon identifier 2517287004]) (see Fig. S3), and all of these genomes were derived from freshwater sediment samples. In several cases (SPGG1, SPGG2, SPGG4, SPGG7, and SPGG9), phylogenetic placement was basal to previously identified taxonomic groups. This basal placement suggests that these genomes represent novel lineages within these phylogenetic groups. Further, the novelty of these lineages supports the hypothesis that these organisms are not remnants of microbial communities exported from the surface ocean. However, differentiating between surface sediment and deep-sea pelagic microorganisms is obfuscated by the limited number of available comparative representatives from deep-sea sources. Functional comparisons between metagenomic samples from various habitats suggest that surface sediment and deep-sea pelagic environments are similar in nature (see Fig. S5), but further studies will be required to elucidate the exchange of organisms and genetic material between these two tightly linked systems.

To determine the relative abundance of the microbial populations, several methods were applied to the data set. Interestingly, the method utilizing conserved markers disagreed with relative abundance estimates derived from previously collected 16S rRNA amplicon data (7) (Fig. 3). Relative to the conserved marker method, the amplicon data appeared to overestimate the abundance of the MG-1 Thaumarchaeota in the sample. This could be the result of biases in the universal primers used to generate the 16S rRNA amplicon data set, as the metagenomic data set was subjected to a randomized linear amplification method for which selection biases should be limited.

In deep-sea sediment environments, microbial metabolisms are determined by the deposition of organic carbon from photosynthetic processes in the surface ocean (44). The input of organic carbon, as determined by the rate of sedimentation and accumulation, is low in the SPG (2). As a result, the microbial biomass in the SPG surface sediments is several orders of magnitude lower than that of other sediments, and metabolic processes are not substantial enough to remove oxygen. However, there is geochemical evidence of microbial respiration, as sediments within the SPG have lower O_2_ concentrations and higher NO_3_^−^ concentrations than bottom seawater (1) (Fig. 1).

The elevated NO_3_^−^ concentration may be attributed to nitrification, the oxidation of ammonium (NH_4_^+^) to nitrate (NO_3_^−^), by chemoautotrophs. Members of the MG-1 Thaumarchaeota are capable of ammonia oxidation to nitrite (NO_2_^−^) (45, 46), the first step in nitrification. This process proceeds via ammonia monooxygenase, which was present in the unbinned Thaumarchaeota from this study. The second step of nitrification is the conversion of NO_2_^−^ to NO_3_^−^, performed by nitrite-oxidizing bacteria (NOB). SPGG3 and SPGG5 belong within the Nitrospinae and Nitrospirae, respectively. Historically, organisms within the phyla Nitrospinae and Nitrospirae have been identified as chemolithoautotrophic NOB. Nitrite oxidation is performed as a reversible reaction via nitrite oxidoreductase (NXR; EC 1.7.99.4) (47). From the recovered portion of the genomes, SPGG3 (Nitrospina) was identified to possess an ortholog of NxrA (see Fig. S4 in the supplemental material), confirming the potential for respiratory nitrite oxidation, and SPGG5 (Nitrospira) contained a putative NxrC homology, suggesting that SPGG5 also is capable of nitrite oxidation but that the other NXR subunits were not recovered. Collectively, these three groups of organisms contain predicted metabolisms that could account for the increase in NO_3_^−^ concentrations with depth in the sediment.

Nitrification is a process linked with autotrophic carbon fixation. The MG-1 Thaumarchaeota are capable of carbon fixation via the 3-OH-prop/4-OH-buty cycle, for which homologs were also identified in the unbinned Thaumarchaeota group. Organisms within the Nitrospinae and Nitrospirae utilize the rTCA cycle to fix carbon. The recovered Nitrospirae genome (SPGG5) possessed the two essential components in the canonical rTCA carbon fixation cycle (39). Pyruvate synthase was not identified within the portion of the SPGG5 genome available, but based on the presence of the essential genes for the rTCA cycle, SPGG5 likely was capable of carbon fixation. There was no evidence of carbon fixation in the recovered SPGG3 genome. Based on the shared traits of the identified Nitrospina species, it remains possible that these genes are present in the unrecovered part of the genome. While SPGG3 had the potential for nitrite oxidation, the current data did not indicate if it shares carbon fixation with the other Nitrospinae (38).

**FIG 5:**
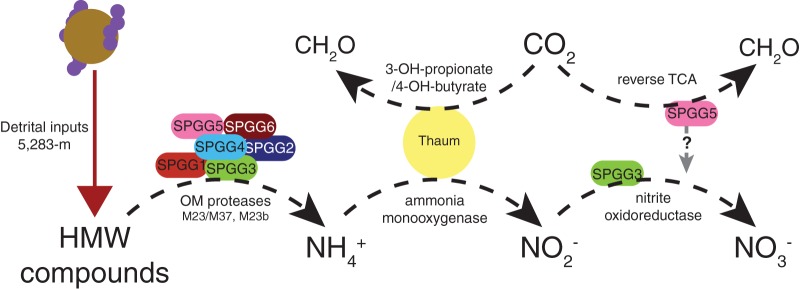
Schematic demonstrating the proposed flow of reduced nitrogen compounds through the microbial community at the sediment-water interface. Black dashed arrows represent proposed biological interactions between microorganisms and the environment. The gray dashed arrow represents unconfirmed biological interaction. Abbreviations: HMW, high molecular weight; OM, outer membrane; Thaum, unbinned Thaumarchaeota; 3-OH-propionate/4-OH-butyrate, 3-hydroxypropionate/4-hydroxybutyrate cycle.

If the unbinned Thaumarchaeota, SPGG3, and SPGG5 are capable of carbon fixation in a carbon/energy-limited environment, this would be a source of locally generated organic carbon, and as such this fixed carbon could serve as a cornerstone for heterotrophic microbial activity. However, this source of “new” chemoautotrophically fixed carbon is linked to organic reduced nitrogen compounds. Like the carbon in the SPG, the source of organic nitrogen compounds is export from the surface ocean, such that autotrophic community members have a limited ability to fix carbon based on the rate of sedimentation. Further, consumption of these compounds via nitrification removes sources of reduced electrons from the environment (Fig. 5). The utilization of reduced compounds as energy sources prevents the utilization of such compounds as biomass. As such, once available nitrogen compounds have been converted to NO_3_^−^, additional metabolisms (e.g., assimilatory nitrate reduction) will be required to convert the oxidized nitrogen to reduced nitrogen suitable for biomass (e.g., proteins and nucleic acids). Therefore, nitrification in the SPG sediment may be predominantly related to energy acquisition, while local carbon fixation may have only a limited impact on stimulating heterotrophic growth due to metabolic inefficiencies in the cycling of organic nitrogen to NO_3_^−^ back to organic nitrogen.

Energy acquisition in this proposed energy-limited environment is not restricted only to nitrification. The SPGGs have other putative metabolisms that can harness other sources of reduced compounds. SPGG4, a novel proteobacterium, has several unique pathways for generating energy from the energy-limited SPG sediments compared to the other SPGGs. SPGG4 possessed a MOB that, based on its position within the molybdopterin tree (Fig. 4), may function as a component in electron transport as an alternative complex III, and, more specifically, in neutrophilic Fe(II) oxidation (41). SPGG4 did not possess homologs of the tetraheme-containing c-type cytochromes, found in the Zetaproteobacteria member M. ferrooxydans PV-1 (41). However, it did contain a diheme c_4_-type cytochrome, without significant identity to the M. ferrooxydans PV-1 cytochromes, which could serve a similar role. It may also be that the requisite cytochromes were present within the missing portion of the SPGG4 genome, or the electron transport chain within SPGG4 may be different from the pathway determined for M. ferrooxydans PV-1.

Microbially mediated neutrophilic Fe(II) oxidation within surface sediments may account for the presence of polymetallic/ferromanganese (FeMn) nodules commonly found to occur along abyssal sediments (48). Fe(II) and Mn(II) may be sourced from distant hydrothermal systems at the East Pacific Rise, as it has recently been quantified that reduced metals from such sources persist for thousands of kilometers with limited removal (49). Previous research has shown FeMn nodules to be associated with uncultured members of the Gammaproteobacteria (7). The process by which FeMn nodules are formed has not been previously identified; however, if SPGG4, a novel proteobacterium, is capable of Fe(II) oxidation, it could offer a source for the Fe(III) oxides in FeMn nodules. Further research will be required, but targeting this novel member of the Proteobacteria from oligotrophic sediments may provide insight into the formation/maintenance of FeMn nodules in the environment.

Additionally, SPGG4, unlike other SPGGs, possessed a potential pathway for the consumption of methanol via formaldehyde to formate. Once methanol is converted to formaldehyde via a putative methanol dehydrogenase, formaldehyde then could react with methanofurans or be processed by a putative quino(hemo)protein alcohol dehydrogenase to generate formate. Methanofurans, commonly associated with methanogenesis, can be generated through a number of biosynthetic pathways and act as coenzymes in processes that oxidize formaldehyde to formate (50, 51). The formate product can then be used as a sole source of carbon and energy. The source of methanol is unclear. As methanol is generally a product of fermentation or the oxidation of methane, both sources would require an anaerobic metabolic process, as abiotic methane production has not been documented; however, the SPG sediments are fully oxygenated. Though not observed, it may be possible that hypoxic/anoxic microenvironments exist within SPG sediments, similar to the case for the surface ocean (52).

Lastly, based on the presence of a putative Ni-Fe hydrogenase, SPGG4 may have been capable of the oxidation of H_2_ to generate cellular energy. The presence of the Ni-Fe hydrogenase is interesting, as a current theory for the SPG is that radiolysis of water via radioactive elements within the sediment could generate molecular H_2_, which could be used as an energy source by microbes (2). This suggests that SPGG4 was capable of utilizing this naturally occurring H_2_ source. Further, H_2_ also can be generated as the result of anaerobic microbial processes, such that, like conversion of methanol to formate, SPGG4 may utilize Ni-Fe hydrogenase to exploit the products of anaerobic microbial metabolisms.

Exploring genomic adaptations proposed to indicate oligotrophic lifestyles may approximate the extent to which microorganisms in the SPG surface sediments experience energy limitation (6). There are several categories that have been suggested to differentiate between oligotrophic and copiotrophic lifestyles: (i) presence/absence of motility mechanisms; (ii) evidence of viral interactions (see Text S1 in the supplemental material); and (iii) compounds targeted by microbial metabolisms (6).

Motility is an energetically expensive process, as is the maintenance of genetic material that does not provide evolutionary benefits. Previous work has shown that the presence of motility genes decreases on a gradient from higher- to lower-energy environment (3, 53). Six of the nine SPGGs possessed genes that grant motility to the organism. Four (SPGG1, -3, -5, and -8) possessed near-complete or complete flagellar biosynthesis operons, while two contained genes required for gliding motility (SPGG4 and -8). The presence of motility genes indicates that these organisms have been traversing from one resource to another, suggesting that there is sufficient energy for metabolic processes and motility. All of the SPGGs that possessed putative flagella also possessed genes that participate in sensing the environment in order to perform chemotaxis. As the energy requirement for motility is high and the SPG sediments are energy poor compared to other sediment environments, the resources reaching the surface sediments may be patchy either spatially or temporally.

Catabolic genes for high-molecular-weight (HMW) compounds were assessed for insight into the available resources of the environment. Six of the SPGGs possessed predicted extracellular, membrane-bound proteases necessary for the extracellular degradation of HMW proteins, although additional proteases without predicted localization may be extracellular in nature (Table 2). Further, the SPGGs possessed ABC-type transporters related to the translocation of peptides and amino acids (see Table S3 in the supplemental material). As ABC-type transporters represent a family of transporters that require ATP to translocate compounds across the cellular membrane, they act as a proxy for determining which compounds organisms are selectively transporting. The degradation of HMW proteins into peptides and amino acids could provide a source for reduced nitrogen compounds, such as ammonium, which in turn could be utilized by the identified nitrifiers (Fig. 5).

The SPGGs were assessed for functional domains that contribute to the degradation of recalcitrant carbon sources. As expected, the SPGGs did not contain putative CDS with predicted function related to the degradation of recalcitrant carbon typically derived from terrestrial sources. SPGGs possessed chitinases and domains related to the extracellular degradation of oligosaccharides and phenolic compounds, specifically laccases, oxygenases, and peroxidases (Table 2). Although they are difficult to access due to partially reconstructed genomes, these features do not appear to be universal for all of the SPGGs, and no single SPGG utilizes a full suite of genes to access all possible recalcitrant carbon compounds. While there may be niche specialization and/or selective forces reducing the number of recalcitrant carbon-reactive genes in each SPGG, it may also indicate that the SPGGs utilize recalcitrant carbon compounds as one source of carbon among a pool of other potential sources. The implication here may be that the microorganisms in the surface sediment beneath oligotrophic ocean waters utilize HMW compounds similar to those used by organisms beneath more productive waters, but that the source of these compounds is more ephemeral and less abundant. If the transient nature of HMW compounds is the prevailing selective evolutionary pressure in this environment, organisms capable of exploiting newly settled sources of organic material via motility and degradation, but also capable of degrading recalcitrant compounds, would be poised to survive during periods of feast or famine.

Even though the SPG sediments are oxic, an examination was performed for genes involved with anaerobic processes. There was no indication of dissimilatory sulfate reduction, Fe(III) reduction, and, with the exception of the FMR dehydrogenase identified in SPGG4, methanogenesis. However, there was an indication for genes related to denitrification, specifically the presence of nitrite reductase (NirK) in one of the novel alphaproteobacterium members (SPGG7) and nitric oxide reductase (NorB) in the unbinned Gammaproteobacteria. Collectively, these processes could reduce NO_2_^−^ to NO to N_2_O through partial denitrification. Denitrification is an anaerobic process that can be present in hypoxic and anoxic environments. This indicates that microenvironments, as in the oxic marine surface waters, exist in the SPG surface sediment where O_2_ concentrations are reduced below the resolution that can be measured through oxygen sensors and interstitial water collection. Further supporting this hypothesis is the presence of the FMR and formate dehydrogenases present in SPGG4 (mentioned above), as well as aerobic and anaerobic formate dehydrogenases in both Alphaproteobacteria (SPGG1 and -7) and one of the novel proteobacteria (SPGG2), as these represent putative genes that can take advantage of organic compounds produced as the result of fermentation. It is possible that these processes are spatially linked in a microenvironment, with nitrification spurring heterotrophic growth resulting in hypoxic conditions, denitrification, and anaerobic fermentation.

The presence of both extracellular proteases and peptide-specific transporters suggests that the SPGGs are utilizing labile HMW compounds as a source of biological components and energy (Fig. 5). Many of the SPGGs possess putative CDS capable of accessing recalcitrant compounds, a trait more common in energy-limited environments. Collectively, it appears that microorganisms that inhabit the SPG surface sediment are poised to gather energy from a variety of reduced compounds, and while this environment is not like the energy-rich coastal sediments, neither is it like oligotrophic environments that are limited to sessile organisms digesting recalcitrant compounds.

### Closing remarks.

The SPG represents one of the most oligotrophic marine environments on earth. This research provides a glimpse into the metabolic potential of microorganisms that inhabit the surface sediments beneath oligotrophic marine environments. Despite substantially lower biomass than that of sediment below more productive waters, the microbes present in the SPG surface sediments have the potential to perform complex transformations of the bioavailable nitrogen and carbon. The data suggest that SPG microbes have the potential to digest HMW compounds and, through key community members, convert this reduced organic nitrogen to nitrate, removing it from the sediment and requiring organisms at deeper depths to generate necessary organic nitrogen via assimilatory nitrate/nitrite reduction (Fig. 5). This evidence suggests that the SPG microbes begin the digestion of recalcitrant compounds, removing another energy source from organisms at deeper depth, and modify the sediment surface environment through the oxidation of reduced iron and/or manganese, likely contributing to the formation of ferromanganese nodules. Based on our current understanding, these transformations may occur in up to one-third of deep-sea sediment environments and may have a significant impact on global biogeochemical cycles.

## Supplementary Material

Supplemental material
