# Supplementary material for: Potential Mechanisms for Microbial Energy Acquisition in Oxic Deep-Sea Sediments
Source: Appl Environ Microbiol. 2016 Jun 30;82(14):4232–43. doi: 10.1128/AEM.01023-16 (PMC4959193; doi:10.1128/AEM.01023-16)
Supplement: Supplemental material [file supp_82_14_4232__index.html]

Supplemental material 

# Potential Mechanisms for Microbial Energy Acquisition in Oxic Deep-Sea Sediments

## Supplemental material

- Supplemental file 1 -

  Supplemental materials and methods (reliability of putative composite genomes), results (porewater geochemistry, binning, phylogenetic assignments, microbial metabolism — phosphorous and vitamin acquisition, evidence of energy limitation? — viral interaction), and discussion; maximum-likelihood phylogenetic tree generated in RAxML, based on the concatenated phylogenetic markers of transcription elongation factor G and ribosomal protein S11 and S13 (825-amino-acid alignment) sequences for 59 microbial genomes, including SPGG8 and SPGG9 (Fig. S1); maximum-likelihood phylogenetic tree generated using PHYML (603-bp alignment) for 52 candidate phylum NC10 16S rRNA sequences, including SPGG6 (Fig. S2); maximum-likelihood phylogenetic tree generated using PHTML (813-bp alignment) for 55 reference 16S rRNA sequences, including SPGG1 and SPGG3 (Fig. S3); identified components of the previous nitrite oxidoreductase alpha subunit (NXR) phylogenomic trees (Fig. S4); results of the HMMER3 v3.1b1 search of putative CDS for the SPG samples and the additional metagenomes against the TIGRFAM v14 database (Fig. S5); presence/absence of ten phylogenetic markers used to construct the full phylogenetic tree (Table S1); list of the reference genes used to construct HMMs and searched using BLASTP within the data set (Table S2); abundance of ABC-type transporter components for each SPGG (Table S3); presence/absence of different phosphorous transporters, vitamin biosynthesis pathways, and vitamin transporters (Table S4); Newick tree format of the RAxML tree shown in Fig. 2 (Data S1).

  PDF, 916K
- Supplemental file 2 -

  RAST annotations used in the analysis of the putative genomes (Data S2).

  XLS, 20M
