# Supplementary material for: Potential Mechanisms for Microbial Energy Acquisition in Oxic Deep-Sea Sediments
Source: Appl Environ Microbiol. 2016 Jun 30;82(14):4232–43. doi: 10.1128/AEM.01023-16 (PMC4959193; doi:10.1128/AEM.01023-16)
Supplement: Supplemental material [file AEM.01023-16_zam999117260so1.pdf]

Potential Mechanisms for Microbial Energy Acquisition in Oxic Deep Sea Sediments

Benjamin J. Tully* and John F. Heidelberg

*Corresponding author

Supplemental Information

Materials and Methods

Reliability of Putative Composite Genomes

CheckM results were assessed independently utilizing a method described in Tully *et al*. (2014). Based on the putative taxonomic assignments of the bins/genomes, finished genomes of the same taxonomic group were accessed from IMG (1) and used to construct reference tables and databases by which to compare core functions of the taxonomic group, through the presence and absence of TIGRFam (2) assignments, and the abundance of taxon specific single copy genes (3, 4) (http://github.com/bjtully/BioData/).

Core functions were determined by comparing the CDS of IMG and putative composite genomes against the TIGRfam database (v 14.0) using hmmscan (parameters: –noali, -E 0.0001, –tblout). An approximation of how completely a putative composite genome represents a member of that taxonomic group (percent completeness) was determined based on the number of shared TIGRfam functions within the IMG finished genomes, and the occurrence of these functions in the putative composite genomes.

Taxon specific single copy genes were identified from the same group of finished genomes. Of the finished genomes, one was selected as the most "phylogenetically-central" based on a neighbor-joining tree (bootstrap = 10) of the DNA gyrase subunit B protein. This phylogenetically-central genome was compared to itself using BLASTP (parameter: -evalue 0.001) and genes with multiple copies within the genome were filtered out based on 80% amino acid identity (AAID) and 80% alignment length. This list of genes was then used to iteratively search the other finished genomes using BLASTP (parameter: -evalue 0.001) with a cutoff of 50% AAID and 80% alignment length. Genes present in multiple copies or without homologs at this cutoff were removed from the list. The final list of genes was BLASTP (parameter: -evalue 0.001) against the putative CDS of each putative composite genome and filtered (50% AAID; 80% alignment length). Taxon specific single copy genes that occurred multiple times within a

80% alignment length). Taxon specific single copy genes that occurred multiple times within a putative composite genome were used to calculate a value that approximates the presence of multiple genomic sources (percent contamination).

Results

Porewater Geochemistry

At SPG site 10, O_2 porewater measurements decreased from the overlaying water concentration of 290 µM to a mean value of 213 µM at 50 cm below the seafloor (cmbsf). Porewater measurements also indicated a sharp decline in the percent amount of total organic carbon (%TOC) from 5 to 40 cmbsf, decreasing from 0.57 %TOC to 0.11 %TOC. Conversely, NO_3^- porewater measurements show a sharp increase from overlying water through the first 5 cmbsf, increasing from 32 µM to 36 µM. However, from 5-45 cmbsf, NO_3^- slightly increases to a maximum of 39 µM.

Binning

The initial ESOM map produced several distinct putative genomic bins, however, as each contig has been assigned a putative taxonomy based on the consensus of LCA assignments of each putative CDS along the contig, it was evident that the general assignment "Proteobacteria" was pervasive throughout the map, without any distinct clustering patterns. Two different ESOM maps were constructed using contigs assigned as "Proteobacteria" (10,155 contigs) and contigs assigned to other taxonomic groups (3,716 contigs). ESOM mapping identified 24 putative genomic bins.

Phylogenetic Assignments

SPGG1 possessed a single 16S rRNA gene copy within the binned genome. This 16S rRNA gene had no matches in the SILVA database (Ref123) and the closest match in the NCBI RefSeq database at 83% nucleotide identity (NAID) was to *Geobacter metallireducens* GS-15 and NCBI NT database at 88% NAID and 73% coverage was an uncultured 16S rRNA clone (JX227029). SPGG3 possessed two copies of the 16S rRNA gene within the binned genome. One copy (1,508 bp) had no matches in the SILVA database and a match in the NCBI NT database at 97% NAID and 29% coverage to a 16S rRNA clone fragment (EU491069). The other copy (1,470 bp) was

assigned matches from the SILVA database to several uncultured *Nitrospina* and a NCBI RefSeq database match at 85% NAID to *Geopsychrobacter electrodiphilus* A1. Because there were no suitable full-length matches for the longer SPGG3 16S rRNA gene, it was not included for phylogenetic tree construction. The shorter SPGG3 16S rRNA gene branches with the uncultured *Nitrospina*. The SPGG1 16S rRNA gene branched with a group of environmental sequences that are divergent from the currently identified phylogenetic groups.

Additionally, SPGG5 contained a copy of the 23S rRNA gene, which was poorly aligned (37% coverage) and 79% identical to *Dehalogenimonas lykanthroporepellens* BL-DC-9, a *Chloroflexi*.

Microbial Metabolism - Phosphorous and Vitamin Acquisition

Based on annotations, means for the acquisition of phosphorous can be identified in all of the SPGGs except two (SPGG6 and -9), the "Unbinned Thaumarchaeota", and "Unbinned Gammaproteobacteria" (Table S4). SPGG3, -5, and the "Unbinned Gammaproteobacteria" contain components of high-affinity phosphate transporters. This is in comparison to SPGG1, -2, -4, and the "Unbinned Gammaproteobacteria" which contain low-affinity phosphate transporters. Additionally, SPGG1, -2, -3, -8, the "Unbinned Thaumarchaeota", and the "Unbinned Gammaproteobacteria" also possess components for phosphonate transport.

The SPGGs were assessed for the presence of vitamin B1 (thiamin), B6 (pyridoxine), and B12 (cobalamin) synthesis and/or transporter components (Table S4). SPGG1, -3, -4, and -5 are capable of the biosynthetic process for generating thiamin, while SPGG1, -6, -7, and the "Unbinned Gammaproteobacteria" possess thiamin transporters. The "Unbinned Thaumarchaeota" are the only group capable of cobalamin production, but both SPGG3 and the "Unbinned Gammaproteobacteria" possess cobalamin transporters. SPGG7 is the only genome capable of pyridoxine synthesis.

Evidence of Energy Limitation? - Viral interaction

The SPGGs were examined for annotated genes that could be used as evidence of viral interactions. Not all SPGGs had annotated evidence. Both SPGG1 and -2 contained annotated phage-related integrase genes, while SPGG1 also contained four putative genes with annotations for phage and prophage repressors. SPGG4 contained four phage related genes, including a phage-associated DNA helicase, a phage capsid gene, and two genes without predicted functions.

The SPGG4 contig that contained the phage-associated DNA helicase had low coding density (56.9%; genome average = 74.4%) and an annotated, putative hypothetical plasmid gene, a hemolysin-type calcium-binding region gene, and an additional 14 hypothetical genes. SPGG5 contained an annotated CRISPR-related Cas protein, but further examination of the contigs did not reveal a predicted CRISPR locus. SPGG6 contained an annotated phage-tail protein clustered near four hypothetical proteins. Further, the "Unbinned Gammaproteobacteria" contained 21 contigs with 27 annotated phage proteins, with various predicted functions, such as tail sheath proteins, shock proteins, a terminase, and a DNA-binding protein.

Discussion

The initial ESOM map contained several clearly defined bins, but overall the map contained a large number of contigs assigned as "Proteobacteria". The distribution of these contigs was evenly spaced across the entire map and, based on an initial assessment of bin integrity, caused putative bins to contain several taxonomic signals. Assignment using the NCBI RefSeq database and a consensus of the LCAs is likely the cause of contigs within bins being assigned to the "Proteobacteria". Several of the SPGGs belong to poorly defined/represented phylogenetic groups. Contigs within in a novel genome with few matches to the NCBI RefSeq database, may become assigned as "Proteobacteria" due to only a few putative CDS. Assignment as "Proteobacteria" is more likely to occur than another taxonomic assignment because a large majority of the genomes in RefSeq belong to the *Proteobacteia*, and predominantly the *Alpha-* and *Gammaproteobacteria*. By splitting the contigs using taxonomic assignment, it was possible to resolve these issues and provide higher resolution binning results. Many of these contigs were never assigned to a putative bin, as even after the iterative mapping, approximately 5,500 contigs remained unassigned.

The robust number of contigs >3 kbp in length allowed for utilization of ESOMs as a way of grouping contigs into putative bin. A majority of the initial bins were determined to contain too few putative CDS and had a limited degree of genomic completeness (generally <700 putative CDS and <30% complete). The largest bins, "Unbinned Thaumarchaeota" and "Unbinned Gammaproteobacteria", had congruent taxonomic assignments amongst the contigs, but, based on counts of conserved markers, clearly contained multiple distinct genomes. However, both bins had the ability to offer information regarding metabolic potential for groups with essential

roles in local biogeochemical cycling, and were used in this capacity.

All but one of the remaining bins were determined to contain >50% of an environmental genome, and all but two had low (<5%) "contamination", representing the portion of the environmental genome that could be derived from other sources but is currently unresolvable. These values suggested that the nine remaining bins were good, draft quality environmental composite genomes and could be used as the near-equivalent of a isolate genome in order to link metabolic function with phylogeny. For the genomes that had >5% contamination, SPGG3 was only slightly above the 5% cutoff (5.13%). SPGG1 was the most complete environmental genome, but also had >10% contamination (11.97%). The overall quality of SPGG1 increases if the calculated value for strain heterogeneity (genes identified as duplicates when considered for contamination, but with >90% amino acid identity (6) is considered, as 26.19% of the contamination value (3.13%) was determined to be derived from closely related strains of same organism. As such, SPGG1 represents a good approximation of this organism within the SPG surface sediment environment, but should not be used as reference in future genomic comparisons.

Three of the other SPGGs (SPGG1, -3, and -6) had rRNA genes within their putative genome. Each of these sequences had poor matches to the genomes within the NCBI RefSeq database (79-85% identity). This low nucleotide identity further supports the novel nature of these lineages amongst previously sequenced and publically available genomes. The placement of the SPGG3 16S rRNA gene amongst the *Nitrospina* was in agreement with the marker gene phylogenetic tree. The placement of the SPGG1 16S rRNA gene did not agree with the marker gene tree, but due to the lack of suitable neighbors and the deep-branching nature of the group amongst the *Alphaproteobacteria* this may likely be attributed to long-branch attraction.

Previous research, examining organisms with oligotrophic and copiotrophic lifestyles (7), had shown that oligotrophs tended to have little to no indication of viral interactions, either defense, such as CRISPRs, or prophage integration. Five (SPGG1, -2, -4, -5, and -6) of the SPGGs and the "Unbinned Gammaproteobacteria" show evidence of viral interactions. Except for SPGG5, which contains a CRISPR-related Cas gene, all of the instances in the SPGGs appear to be indicative of prophage integration. It is understood that there is an interaction between viral attack, cellular abundance, and energy-limitation. The presence of phage genes within the SPGGs suggests that organisms in the surface sediment are undergoing infection, lysis, and

prophage integration similar to other copiotrophic organisms and energy replete environments.

The microbes in the SPG surface sediment utilize a variety of mechanisms to access bioavailable phosphorous. Phosphate (PO_4^{2-}) concentrations for the sediment were measured at 1.78 μM at 5-15 cmbsf (8). There appears to be a stark differentiation between several of the SPGGs; SPGG3 and -5, both putatively nitrite-oxidizing bacteria, possessed putative high-affinity phosphate transporters, compared to SPGG1, -2, -4 and -7, which possessed putative low-affinity phosphate transporters. These differences could be an indication of the phosphorous demand for these organisms, potentially attributed to the shared metabolic capability of SPGG3 and -5, relative to the other organisms. Additionally, SPGG1, -2, -3, and -8 possessed phosphonate transporters which can be used to supplement organic phosphorous demands, a trait that may account for SPGG1 and -2 having possessed low-affinity transporters, so that both sets of transporters can collectively provide sufficient PO_4^{2-} for biosynthesis.

Mechanisms related to the biosynthesis of vitamin B1, B6, and B12 were identified in the SPGGs. For vitamin B1 (thiamin), it appears as if SPGG1, -3, -4, and -5 were prototrophs, capable of satisfying biological needs of thiamin via biosynthesis. Interestingly, SPGG1 possessed a thiamin transporter, like SPGG6 and -7. However, based on the genomic results, SPGG6 and -7 appear to be auxotrophs and cannot synthesize their own thiamin. It is possible that SPGG1 may have supplied thiamin to SPGG6 and -7, providing some basis of cooperation between microbial groups. A similar dynamic was possible for vitamin B12 (cobalamin), whereby the "Unbinned Thaumarchaeota" appeared to be a source of cobalamin in the environment, but that SPGG3 and the "Unbinned Gammaproteobacteria" could utilize this source, either via some undetected transporter or after cell lysis. Interestingly, only SPGG7 appeared to have the capacity to synthesize vitamin B6 (pyridoxine). The available genomic regions of the other SPGGs did not possess annotated transporters for pyridoxine export or consumption.

Supplemental Material Legends

Table S1. Presence/absence of ten phylogenetic markers used to construct the full phylogenetic tree.

Table S2. A list of the reference genes used to construct HMMs and searched using BLASTP within the dataset.

Table S3. Abundance of ABC-type transporter components (including permease, ATP-binding,

and peptide transport are denoted with black circles.

STable 4. Presence/absence of different phosphorous transporters, vitamin biosynthesis pathways, and vitamin transporters.

Data S1. Newick format of the RAxML tree present in Figure 2 of the main manuscript.

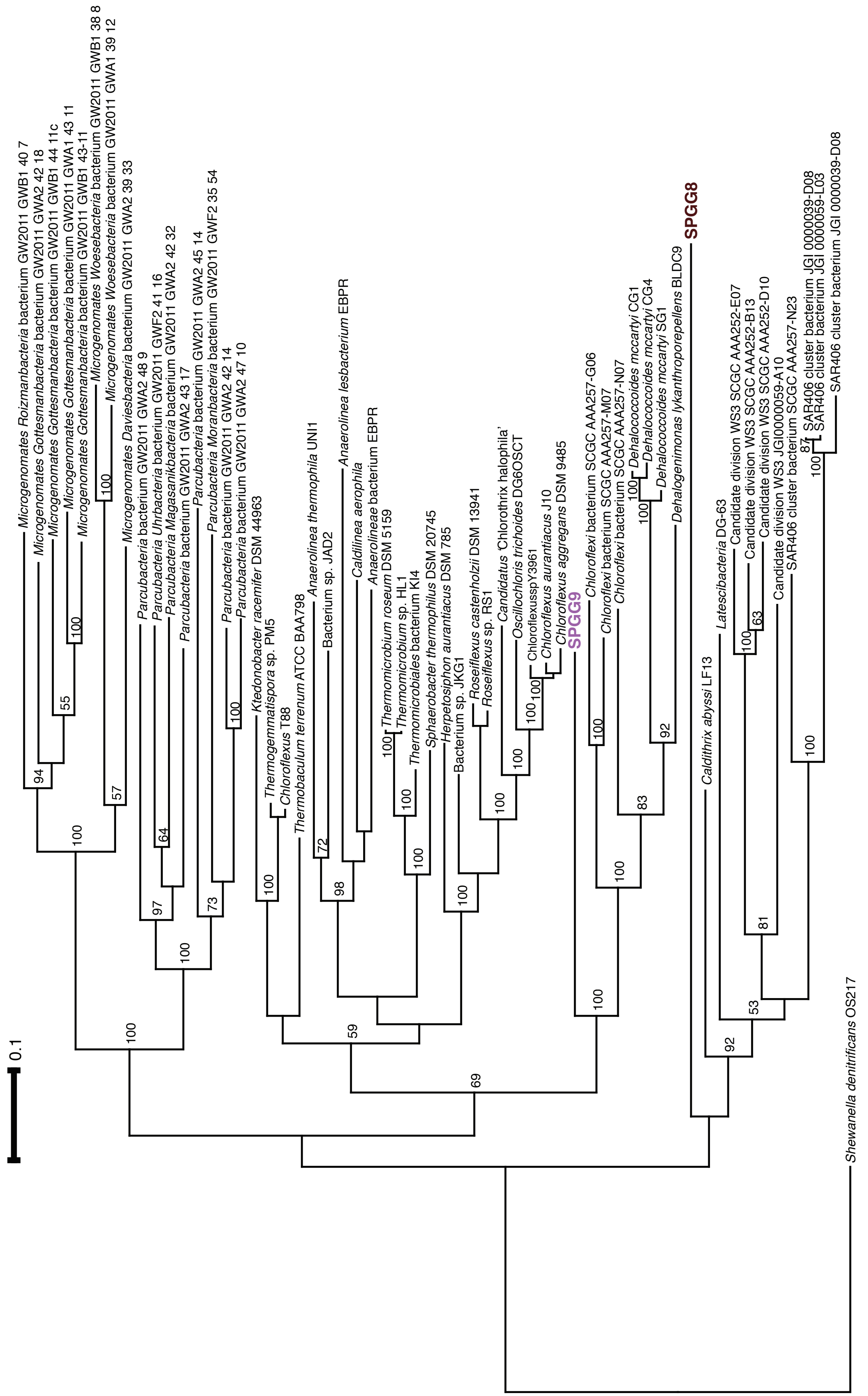

Figure S1. A maximum likelihood phylogenetic tree generated in RAxML based on the concatenated phylogenetic markers of transcription elongation factor G, and ribosomal proteins S11 and S13 (825 amino acid alignment) sequences for 59 microbial genomes, including SPGG8 and SPGG9 (Bootstraps = 100). Only bootstrap values ≥50 are shown.

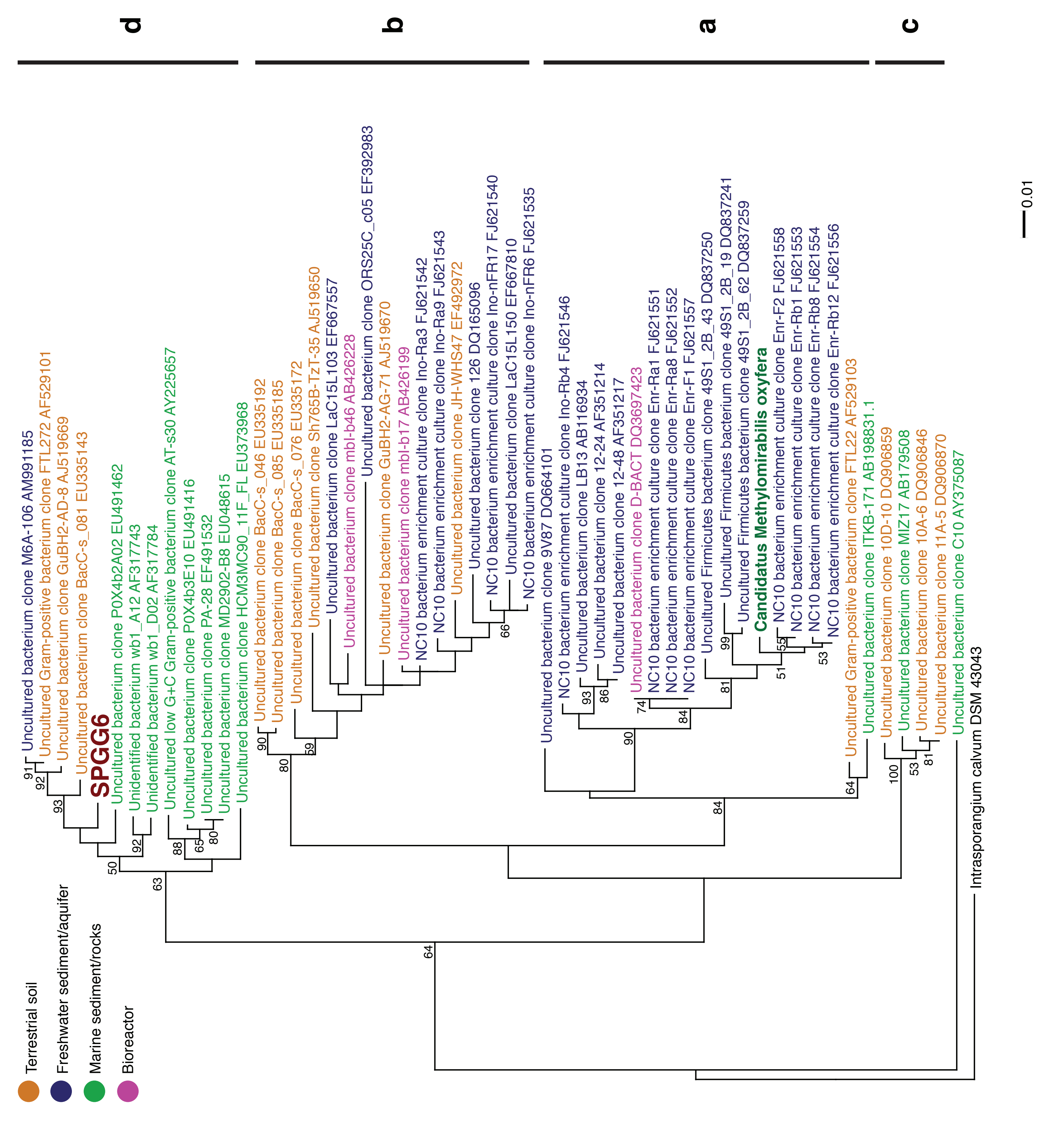

Figure S2. A maximum likelihood phylogenetic tree generated using PHYML (603 bp alignment) for 52 Candidate Phylum NC10 16S rRNA sequences, including SPGG6 (Bootstraps = 100). Only bootstrap values ≥50 are shown.

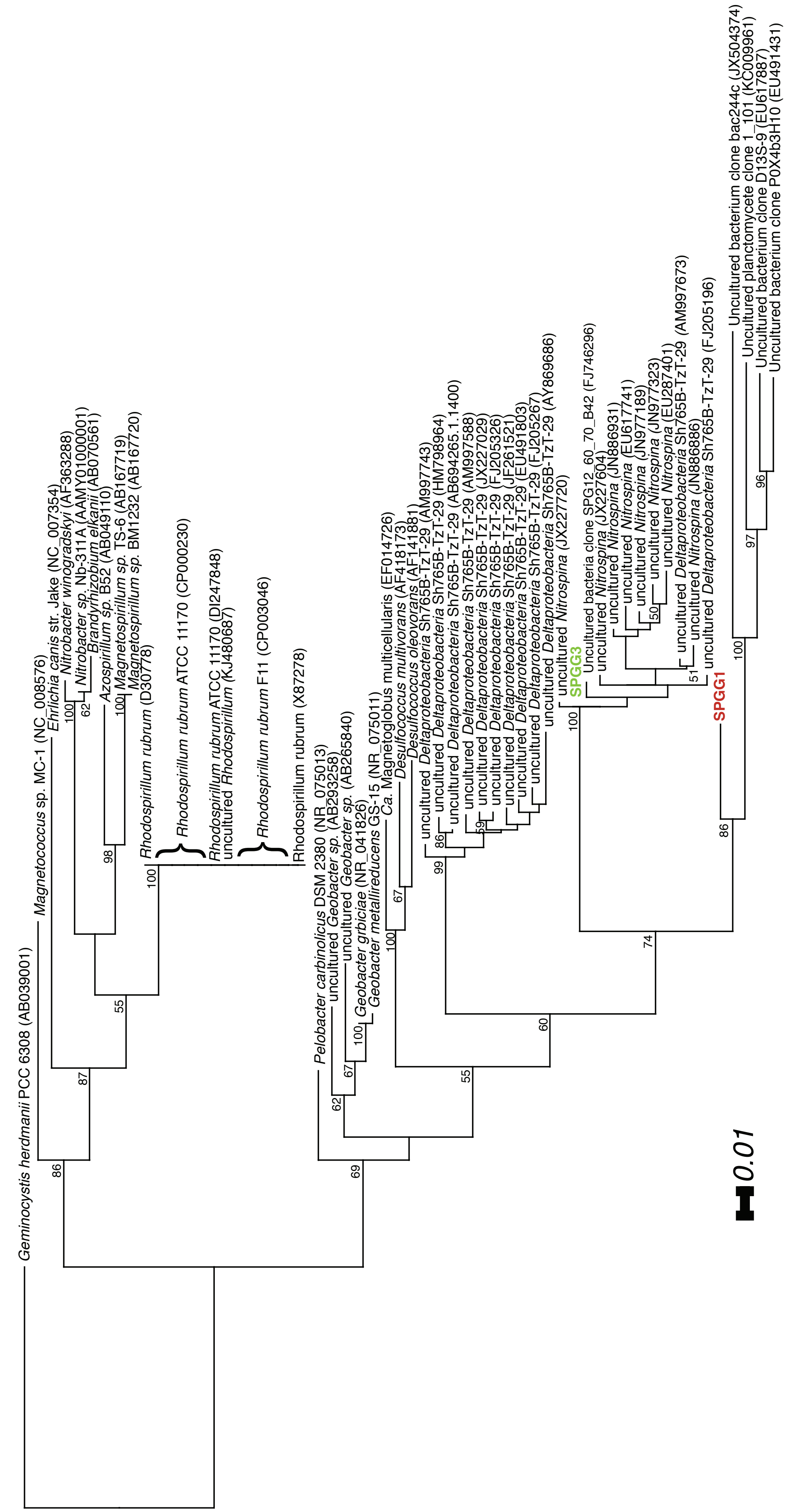

Figure S3. A maximum likelihood phylogenetic tree generated using PHYML (813 bp alignment) for 55 reference 16S rRNA sequences, including SPGG1 and SPGG3 (Bootstraps = 100). Only bootstrap values ≥50 are shown.

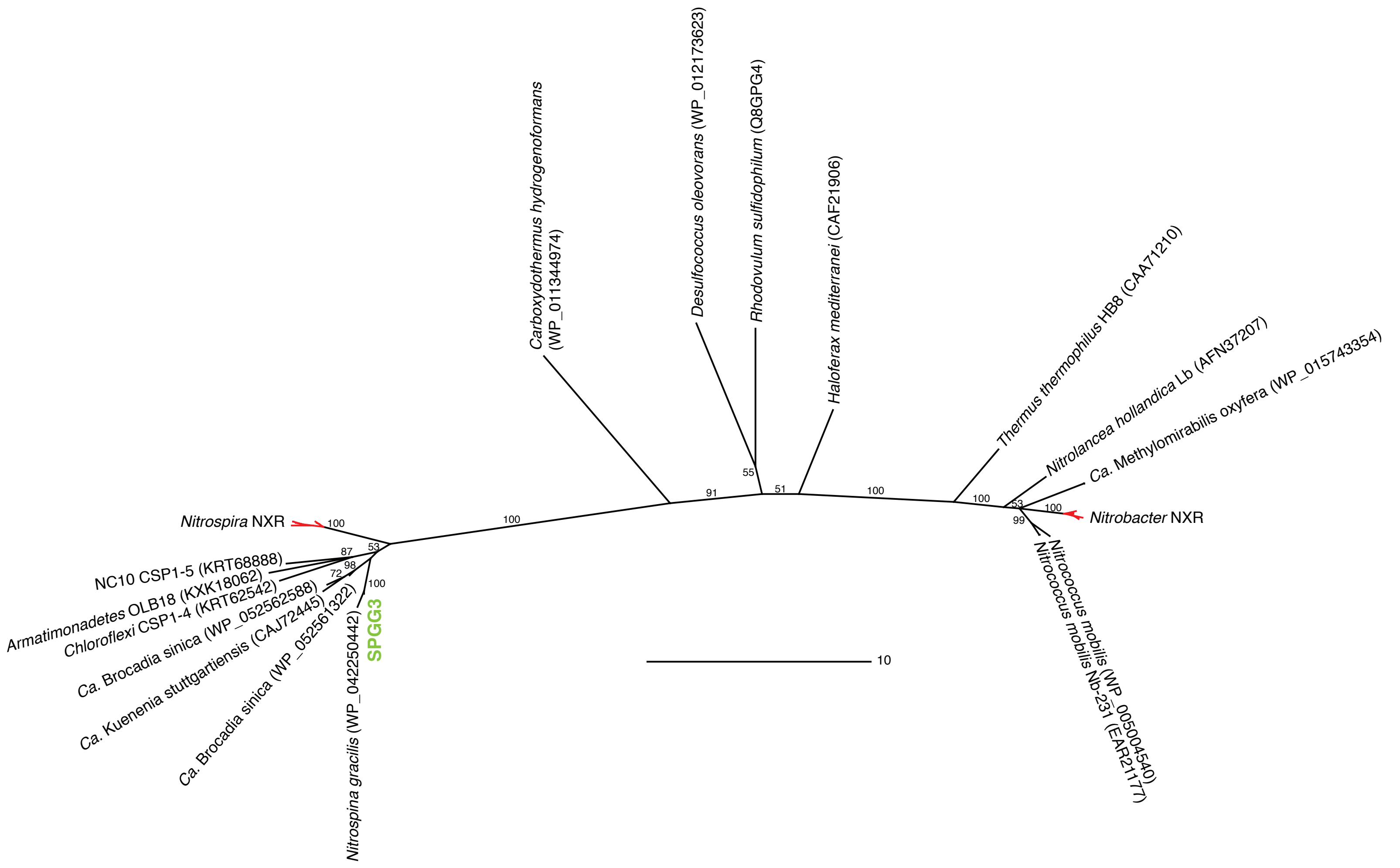

Figure S4. Identified components of the previous nitrite oxidoreductase alpha subunit (NXR) phylogenomic trees, as in Lücker *et al.* (9) and Daims *et al.* (10). Proteins were aligned using MUSCLE (parameters: default; 253 amino acid alignment) and used to construct a Neighbor-Joining tree with 31 sequences and the putative NXR from SPGG3 (parameters: model, Jukes-Cantor; bootstraps, 100). Sequences included in "Nitrobacteria NXR": *N. hamburgensis* (WP_041359063, WP_011511827, WP_011509502), *N. winogradskyi* (WP_011314088, WP_011315305), *N.* sp. Nb-311A (WP_009800186, WP_040675862). Sequences included in "Nitrospira NXR": *N. moscoviensis* (WP_053381686), *Ca.* N. inopinata (WP_062483509), *N. defluvii* (WP_013249749), *Ca.* N. nitrosa (CUS31266), *Ca.* N. nitrificans (CUS36764, CUS38776).

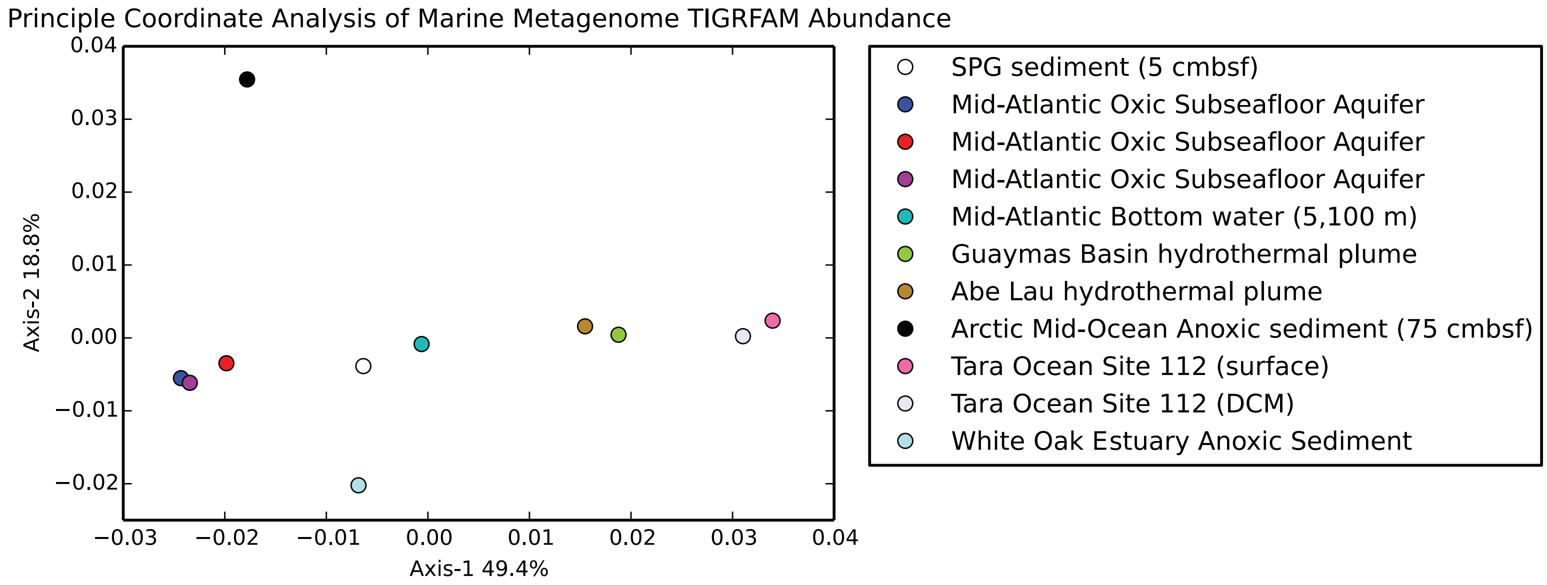

Figure S5. As in Meyer *et al.* (11), putative CDS for the SPG samples and the additional metagenomes were searched using HMMER3 v3.1b1 against the TIGRFAM v14 database (hmmsearch, parameters: -E 0.00001). From the hmmsearch results, putative CDS were assigned to TIGRFAM roles based on the best match. For each metagenome, the fraction for each TIGRFAM role was determined using the total number of TIGRFAM assignments for each sample (no. of putative CDS assigned to a specific role ÷ total no. of putative CDS assigned to all TIGRFAM roles). The normalized fraction of the 116 identified TIGRFAM roles for each sample was visualized using principal component analysis (PCA) to determine the relationship between samples. PCA was performed using the Python library sklearn v0.16.1. Values underwent dimensionality reduction, while being fit to the model. Putative protein sequences were collected from project pages: Mid-Atlantic Samples, GenBank BioProject PRJNA280201; Guaymas Basin hydrothermal plume, IMG Taxon Object ID 3300001683; Abe Lau hydrothermal plume, IMG Taxon Object ID 3300001681; Arctic Mid-Ocean anoxic sediments, DDBJ/EMBL/GenBank Accession LAZR00000000; Tara Ocean Project Site 112 Surface water, EBI Analysis Accession ERZ097110; Tara Ocean Project Site 112 Deep chlorophyll max (DCM), EBI Analysis Accession ERZ097142; White Oak Estuary Anoxic Sediment, GenBank BioProject PRJNA270657.

References

1. V. M. Markowitz, I. M. Chen, K. Palaniappan, K. Chu, E. Szeto, M. Pillay, A. Ratner, J. Huang, T. Woyke, M. Huntemann, I. Anderson, K. Billis, N. Varghese, K. Mavromatis, A. Pati, N. N. Ivanova, and N. C. Kyrpides. (2014) IMG 4 version of the integrated microbial genomes comparative analysis system. *Nucleic Acids Res*, 42:D560–7.

2. D.H. Haft, J.D. Selengut, and O. White. (2003) The TIGRFAMs database of protein families. *Nucleic Acids Research*, 31(1):371–373.

3. B.J. Tully, R. Sachdeva, K.B. Heidelberg, and J.F. Heidelberg. (2014) Comparative genomics of planktonic Flavobacteriaceae from the Gulf of Maine using metagenomic data. *Microbiome*, 2(1):34.

4. P. Engel, V.G. Martinson, and N.A. Moran. (2012) Functional diversity within the simple gut microbiota of the honey bee. *Proceedings of the National Academy of Sciences*, 109(27):11002–11007.

5. R.C. Edgar. (2004) MUSCLE: multiple sequence alignment with high accuracy and high throughput. *Nucleic Acids Research*, 32(5):1792–1797.

6. D. H. Parks, M. Imelfort, C. T. Skennerton, P. Hugenholtz, and G. W. Tyson. (2015) CheckM: assessing the quality of microbial genomes recovered from isolates single cells, and metagenomes. *Genome Research*, 25(7):1043–1055.

7. F.M. Lauro, D. McDougald, T. Thomas, T.J. Williams, S. Egan, S. Rice, M.Z. DeMaere, L. Ting, H. Ertan, J. Johnson, S. Ferriera, A. Lapidus, I. Anderson, N. Kyrpides, A.C. Munk, C. Detter, C.S. Han, M.V. Brown, F.T. Robb, S. Kjelleberg, and R. Cavicchioli. (2009) The genomic basis of trophic strategy in marine bacteria. *Proceedings of the National Academy of Sciences*, 106(37):15527–15533.

8. D'Hondt, S., Inagaki, F., Alvarez Zarikian, C.A., and the Expedition 329 Scientists. (2011) Site U1369. In *Proceedings of the IODP*. International Ocean Discovery Program (IODP).

9. S. Lücker, M. Wagner, F. Maixner, E. Pelletier, H. Koch, B. Vacherie, T. Rattei, J.S.S. Damste, E. Spieck, D. Le Paslier, and H. Daims. (2010) A Nitrospira metagenome illuminates the physiology and evolution of globally important nitrite-oxidizing bacteria. *Proceedings of the National Academy of Sciences*, 107(30):13479–13484.

10. H. Daims, E. V. Lebedeva, P. Pjevac, P. Han, C. Herbold, M. Albertsen, N. Jehmlich, M. Palatinszky, J. Vierheilig, A. Bulaev, R.H. Kirkegaard, M. von Bergen, T. Rattei, B. Bendinger, P.H. Nielsen, M. Wagner (2015). Complete nitrification by Nitrospira bacteria. *Nature*, *528*(7583), 504–509.

11. J. L. Meyer, U. Jaekel, B. Tully, B. T. Glazer, C. G. Wheat, H. Lin, C. Hsieh, J. P. Cowen, S. M. Hulme, P. R. Girguis, and J. A. Huber. (2016) A distinct and active bacterial community in cold oxygenated fluids circulating beneath the western flank of the Mid-Atlantic ridge. *Scientific Reports*, 6:22541

Table S1.

Order of alignment arrangement										
				Ribosomal Proteins						
**Genome ID**	**DNA gyrase subunit B**	**DNA-directed RNA polymerase subunit B**	**Transcription elongation factor - G**	**RpL2**	**RpL4**	**RpL7**	**RpL11**	**RpS11**	**RpS13**	**RpS19**
SPGG1	+	-	+	+	+	-	-	+	+	+
SPGG2	+	+	+	-	-	+	+	+	+	-
SPGG3	+	+	-	+	+	-	-	+	+	+
SPGG4	+	-	+	+	+	-	-	+	+	+
SPGG5	-	+	-	+	+	+	+	+	+	+
SPGG6	+	+	+	-	-	+	+	-	-	-
SPGG7	+	+	-	+	+	+	+	+	+	+
SPGG8	+	-	+	+	+	-	-	+	+	+
SPGG9	-	-	+	-	-	-	-	+	+	-

Table S2.

**Genes Used to Construct HMMs**		
**GenBank Nucleotide ID/Accession Number**	**Locus & Gene Annotation**	**Source Organism**
**MtrA**		
637679870	Daro_1402 Doubled CXXCH motif	Dechloromonas aromatica RCB: NC_007298
646690176	Slit_2497 decaheme c-type cytochrome, DmsE family	Sideroxydans lithotrophicus ES-1 chromosome: NC_013959
2523654184	D466DRAFT_2462 hypothetical protein	Thiomonas sp. FB-6, DSM 25805 : D466DRAFT_scaffold00001.1
637821629	amb3017 Cytochrome c-type protein nrfB precursor	Magnetospirillum magneticum AMB-1: NC_007626
637918906	Rfer_4082 hypothetical protein	Rhodoferax ferrireducens T118: NC_007908
2508594361	BurJ1DRAFT_0793 decaheme c-type cytochrome, DmsE family	Burkholderiales sp. JOSHI_001 : BurJ1DRAFT_BJB.1
648727851	Sbal175DRAFT_1862 decaheme c-type cytochrome, DmsE family	Shewanella baltica BA175 ctg641: NZ_AEDE01000003
2507443576	Sbal625_1638 decaheme c-type cytochrome, DmsE family	Shewanella baltica OS625 : Sbal625_Contig118.1
648703889	Sbal183DRAFT_1150 decaheme c-type cytochrome, DmsE family	Shewanella baltica OS183 ctg590: NZ_AECY01000002
641287883	Sbal195_1611 hypothetical protein	Shewanella baltica OS195: NC_009997
640829773	Shew185_1577 cytochrome C family protein	Shewanella baltica OS185: NC_009665
640118804	Sbal_1588 cytochrome C family protein	Shewanella baltica OS155: NC_009052
651218391	Sbal117_1700 decaheme c-type cytochrome, DmsE family	Shewanella baltica OS117: CP002811
643461653	Sbal223_2766 cytochrome C family protein	Shewanella baltica OS223: NC_011663
637343663	mtrA decaheme cytochrome c MtrA	Shewanella oneidensis MR-1: NC_004347
638119146	Shewmr4_2511 cytochrome C family protein	Shewanella sp. MR-4: NC_008321
639727283	Shewana3_2677 cytochrome C family protein	Shewanella sp. ANA-3 chromosome 1: NC_008577
638123257	Shewmr7_2579 cytochrome C family protein	Shewanella sp. MR-7: NC_008322
651658092	SOHN41_01519 periplasmic decaheme cytochrome c, MtrA	Shewanella sp. HN-41 44_18: NZ_AFOZ01000018
639816717	Sputw3181_2624 cytochrome C family protein	Shewanella sp. W3-18-1: NC_008750
2510455481	Sput200_1487 periplasmic decaheme cytochrome c, MtrA	Shewanella putrefaciens 200 (Missing data) : CP002457
2519278318	CN32_1477 periplasmic decaheme cytochrome c, MtrA	Shewanella putrefaciens CN-32 : Shewanella
648212711	Fbal_1356 decaheme c-type cytochrome, DmsE family	Ferrimonas balearica DSM 9799 chromosome: NC_014541
2523949880	G506DRAFT_01773 hypothetical protein	Ferrimonas senticii DSM 18821 : G506DRAFT_scaffold00005.5
2509058136	EcthaDRAFT_2766 decaheme c-type cytochrome, DmsE family	Ectothiorhodospira haloalkaliphila Imhoff 51/7, ATCC 51935 : EcthaDRAFT_EHB.4
2507116148	Thimo_2812 decaheme c-type cytochrome, DmsE family	Thioflavicoccus mobilis 8321 : Thimo_Contig50
2523864546	F799DRAFT_03026 hypothetical protein	Rubrivivax benzoatilyticus ATCC BAA-35 : F799DRAFT_scaffold00017.17
640551111	Gura_3626 cytochrome C family protein	Geobacter uraniumreducens Rf4: NC_009483
638463626	Dace_1556 cytochrome c family protein	Desulfuromonas acetoxidans DSM 684, unfinished sequence: NZ_AAEW02000006
**MtoA**		
gi|71907034|ref|YP_284621.1|	double CXXCH motif-containing protein	Dechloromonas aromatica RCB
gi|302879210|ref|YP_003847774.1|	decaheme c-type cytochrome, DmsE family	Gallionella capsiferriformans ES-2
gi|291614952|ref|YP_003525109.1|	decaheme c-type cytochrome, DmsE family	Sideroxydans lithotrophicus ES-1
gi|375104156|ref|ZP_09750417.1|	decaheme c-type cytochrome, DmsE family	Burkholderiales bacterium JOSHI_001
gi|89902840|ref|YP_525311.1|	hypothetical protein Rfer_4082	Rhodoferax ferrireducens T118
gi|470172740|ref|YP_007553626.1|	decaheme c-type cytochrome, DmsE family	Azoarcus sp. KH32C
gi|479299058|gb|ENO87158.1|	decaheme c-type cytochrome, DmsE family protein	Thauera linaloolentis 47Lol = DSM 12138
gi|470169703|ref|YP_007550589.1|	decaheme c-type cytochrome, DmsE family	Azoarcus sp. KH32C
gi|83312116|ref|YP_422380.1|	cytochrome c-type protein nrfB precursor	Magnetospirillum magneticum AMB-1
gi|119774350|ref|YP_927090.1|	decaheme cytochrome c MtrD	Shewanella amazonensis SB2B
**MtrB**		
637470743	rpt2T07 outer membrane protein precursor	Vibrio vulnificus YJ016 chromosome II: NC_005140
649922346	VVM_01306 outer membrane protein, MtrB	Vibrio vulnificus MO6-24/O chromosome chromosome II: NC_014966
637363602	VV20135 hypothetical protein	Vibrio vulnificus CMCP6 chromosome II: NC_004460
637398195	VP1218 putative outer membrane protein precursor	Vibrio parahaemolyticus RIMD 2210633 chromosome I: NC_004603
641033642	A79_1921 outer membrane protein	Vibrio parahaemolyticus AQ3810, unfinished sequence: NZ_AAWQ01000049
648305439	VIPARAQ4037_2777 decaheme-associated outer membrane protein, MtrB/PioB family	Vibrio parahaemolyticus AQ4037 gcontig_1113976698134: NZ_ACFN01000095
646406875	VEA_003778 outer membrane protein	Vibrio sp. Ex25 chromosome 1: NC_013456
639715341	AHA_2766 outer membrane protein	Aeromonas hydrophila subsp. hydrophila ATCC 7966: NC_008570
648703888	Sbal183DRAFT_1149 decaheme-associated outer membrane protein, MtrB/PioB family	Shewanella baltica OS183 ctg590: NZ_AECY01000002
648727850	Sbal175DRAFT_1861 decaheme-associated outer membrane protein, MtrB/PioB family	Shewanella baltica BA175 ctg641: NZ_AEDE01000003
640829772	Shew185_1576 outer membrane protein precursor MtrB	Shewanella baltica OS185: NC_009665
2507443575	Sbal625_1637 decaheme-associated outer membrane protein, MtrB/PioB family	Shewanella baltica OS625 : Sbal625_Contig118.1
640118803	Sbal_1587 outer membrane protein precursor MtrB	Shewanella baltica OS155: NC_009052
651218390	Sbal117_1699 decaheme-associated outer membrane protein, MtrB/PioB family	Shewanella baltica OS117: CP002811
643461654	Sbal223_2767 outer membrane protein precursor MtrB	Shewanella baltica OS223: NC_011663
638119147	Shewmr4_2512 outer membrane protein precursor MtrB	Shewanella sp. MR-4: NC_008321
638123258	Shewmr7_2580 outer membrane protein precursor MtrB	Shewanella sp. MR-7: NC_008322
639727284	Shewana3_2678 outer membrane protein precursor MtrB	Shewanella sp. ANA-3 chromosome 1: NC_008577
637343662	mtrB outer membrane protein precursor MtrB	Shewanella oneidensis MR-1: NC_004347
651658091	SOHN41_01518 outer membrane protein, MtrB	Shewanella sp. HN-41 44_18: NZ_AFOZ01000018
639816718	Sputw3181_2625 outer membrane protein precursor MtrB	Shewanella sp. W3-18-1: NC_008750
2510455482	Sput200_1486 outer membrane protein, MtrB	Shewanella putrefaciens 200 (Missing data) : CP002457
2519278317	CN32_1476 outer membrane protein, MtrB	Shewanella putrefaciens CN-32 : Shewanella
643426644	swp_3280 Outer membrane protein precursor MtrB	Shewanella piezotolerans WP3: NC_011566
637679871	Daro_1403 hypothetical protein	Dechloromonas aromatica RCB: NC_007298
648149490	Galf_2003 decaheme-associated outer membrane protein, MtrB/PioB family	Gallionella capsiferriformans ES-2 chromosome: NC_014394
2515876849	B145DRAFT_03135 decaheme-associated outer membrane protein, MtrB/PioB family	Uliginosibacterium gangwonense DSM 18521 : B145DRAFT_scaffold_9.10
646692265	Nhal_1654 hypothetical protein	Nitrosococcus halophilus Nc4 chromosome: NC_013960
639856855	Hhal_2380 hypothetical protein	Halorhodospira halophila SL1: NC_008789
2518923848	F464DRAFT_05422 decaheme-associated outer membrane protein, MtrB/PioB family	Azoarcus toluclasticus ATCC 700605 : F464DRAFT_scaffold00031.31
**MtrC**		
641287884	Sbal195_1612 hypothetical protein	Shewanella baltica OS195: NC_009997
2507443577	Sbal625_1639 decaheme c-type cytochrome, OmcA/MtrC family	Shewanella baltica OS625 : Sbal625_Contig118.1
641287884	Sbal195_1612 hypothetical protein	Shewanella baltica OS195: NC_009997
648703890	Sbal183DRAFT_1151 decaheme c-type cytochrome, OmcA/MtrC family	Shewanella baltica OS183 ctg590: NZ_AECY01000002
648727852	Sbal175DRAFT_1863 decaheme c-type cytochrome, OmcA/MtrC family	Shewanella baltica BA175 ctg641: NZ_AEDE01000003
640829774	Shew185_1578 decaheme cytochrome c	Shewanella baltica OS185: NC_009665
640118805	Sbal_1589 decaheme cytochrome c	Shewanella baltica OS155: NC_009052

651218392	Sbal117_1701 decaheme c-type cytochrome, OmcA/MtrC family	Shewanella baltica OS117: CP002811
643461652	Sbal223_2765 decaheme cytochrome c	Shewanella baltica OS223: NC_011663
638123256	Shewmr7_2578 decaheme cytochrome c	Shewanella sp. MR-7: NC_008322
639727282	Shewana3_2676 decaheme cytochrome c	Shewanella sp. ANA-3 chromosome 1: NC_008577
639816716	Sputw3181_2623 decaheme cytochrome c	Shewanella sp. W3-18-1: NC_008750
2510455480	Sput200_1488 surface localized decaheme cytochrome c lipoprotein, MtrC	Shewanella putrefaciens 200 (Missing data) : CP002457
2519278319	CN32_1478 outer membrane decaheme cytochrome c lipoprotein, MtrC	Shewanella putrefaciens CN-32 : Shewanella
637343664	omcB decaheme cytochrome c	Shewanella oneidensis MR-1: NC_004347
638119145	Shewmr4_2510 decaheme cytochrome c	Shewanella sp. MR-4: NC_008321
640932391	Ssed_1525 decaheme cytochrome c	Shewanella sediminis HAW-EB3: NC_009831
2524202114	Sfri_2637 extracellular iron oxide respiratory system surfacedecaheme cytochrome c component MtrC	Shewanella frigidimarina NCIMB 400 : Shewanella
2523117992	G505DRAFT_01572 decaheme c-type cytochrome, OmcA/MtrC family	Ferrimonas futtsuensis DSM 18154 : G505DRAFT_scaffold00006.6
2523949881	G506DRAFT_01774 hypothetical protein	Ferrimonas senticii DSM 18821 : G506DRAFT_scaffold00005.5
639755619	Ppro_1575 cytochrome c family protein	Pelobacter propionicus DSM 2379: NC_008609
640822229	SUN_1068 hypothetical protein	Sulfurovum sp. NBC37-1: NC_009663
**Fungal Laccase**		
sp|A2XCN6|LAC18_ORYSI	Putative laccase-18	Oryza sativa subsp. indica
sp|Q2R0L2|LAC19_ORYSJ	Laccase-19	Oryza sativa subsp. japonica
sp|Q6ID18|LAC10_ARATH	Laccase-10	Arabidopsis thaliana
sp|Q0DHL5|LAC11_ORYSJ	Putative laccase-11	Oryza sativa subsp. japonica
sp|Q9LYQ2|LAC13_ARATH	Laccase-13	Arabidopsis thaliana
sp|Q84J37|LAC15_ARATH	Laccase-15	Arabidopsis thaliana
sp|Q7XE50|LAC16_ORYSJ	Putative laccase-16	Oryza sativa subsp. japonica
sp|Q9LMS3|LAC1_ARATH	Laccase-1	Arabidopsis thaliana
sp|Q12542|LAC2_AGABI	Laccase-2	Agaricus bisporus
sp|Q70KY3|LAC1_MELAO	Laccase-1	Melanocarpus albomyces
sp|P17489|LAC1_EMENI	Laccase-1	Emericella nidulans strain FGSC A4
**Fungal Manganese Peroxidase**		
sp|Q02567|PEM1_PHACH	Manganese peroxidase 1	Phanerochaete chrysosporium
sp|P19136|PEM4_PHACH	Manganese peroxidase H4	Phanerochaete chrysosporium
sp|P78733|PEM3_PHACH	Manganese peroxidase H3	Phanerochaete chrysosporium
sp|Q70LM3|PEM2_PHLRA	Manganese peroxidase 2	Phlebia radiata
sp|Q96TS6|PEM3_PHLRA	Manganese peroxidase 3	Phlebia radiata
**Neutrophilic Iron Oxidizing Molybdopterin Oxidoreductase**		
gi|302877693|ref|YP_003846257.1|	molybdopterin oxidoreductase	Gallionella capsiferriformans ES-2
gi|291613111|ref|YP_003523268.1|	molybdopterin dinucleotide-binding region	Sideroxydans lithotrophicus ES-1
gi|389699394|ref|ZP_10184967.1|	anaerobic dehydrogenase, typically selenocysteine-containing	Leptothrix ochracea L12
ZP_01451016	Molybdopterin oxidoreductase Fe4S4 region	Mariprofundus ferrooxydans PV-1
gi|148264818|ref|YP_001231524.1|	nitrate reductase	Geobacter uraniireducens Rf4
gi|404496667|ref|YP_006720773.1|	menaquinol oxidoreductase complex ACIII, molybdopterin-binding-like subunit ActB1	Geobacter metallireducens GS-15
**Magnetosome Operon (MamABCDEFKMOS)**		
mamADEOS	various genes	Magnetospirillum sp. SO-1
mamADEFKMOS	various genes	Magnetospirillum magneticum AMB-1
mamABCDEFKMOS	various genes	Magnetospirillum magnetotacticum MS-1
mamAM	various genes	alpha proteobacterium LEMS
mamABCDFO	various genes	Magnetospirillum gryphiswaldense MSR-1
mamABCEMO	various genes	Magnetovibrio blakemorei
mamBCDEFKMS	various genes	Magnetospirillum gryphiswaldense
mamBCKMS	various genes	Magnetococcus marinus MC-1
mamBK	various genes	alpha proteobacterium CC-2
mamKO	various genes	alpha proteobacterium CB-1
mamK	various genes	gamma proteobacterium SS-5
**Genes Used to Search for Metabolisms of Interest**		
**KEGG Organism Abbreviation/Accession Number**	**Gene Locus Identifier**	**Gene Annotation**
**Calvin Cycle**		
dhy	DESAM_22949	Ribulose bisphosphate carboxylase (EC:4.1.1.39)
smx	SM11_pD1412	putative ribulose-1,5-bisphosphate carboxylase large subunit protein
smi	BN406_06465	ribulose bisphosphate carboxylase large subunit (EC:4.1.1.39)
rpm	RSPPHO_03165	Ribulose bisphosphate carboxylase (EC:4.1.1.39)
mcj	MCON_3086	ribulose bisphosphate carboxylase, large chain (form II)
ali	AZOLI_p10724	Ribulose bisphosphate carboxylase large chain (RuBisCO large subunit)
**Denitrification**		
cps	CPS_0601	nitric-oxide reductase, B subunit (EC:1.7.99.7)
lpa	lpa_03215	nitric-oxide reductase, cytochrome b-containing subunit I (EC:1.7.2.5)
mah	MEALZ_1161	nitric oxide reductase subunit B
cpg	Cp316_0140	Nitric-oxide reductase, cytochrome b-containing subunit I
pau	PA14_06650	c-type cytochrome
pau	PA14_06750	nitrite reductase
mhc	MARHY3064	cytochrome oxidase (EC:1.7.2.1 1.7.99.1)
mhc	MARHY3071	nitrite reductase (EC:1.7.2.1)
reh	H16_B2277	cytochrome cd1 nitrite reductase (NirS) (EC:1.7.2.1)
reh	H16_B2284	C-type cytochrome (NirN)
gau	GAU_2766	putative nitrite reductase (EC:1.7.2.1)
gtn	GTNG_0650	nitrite reductase
pgl	PGA2_239p1440	copper-containing nitrite reductase NirK (EC:1.7.2.1)
bra	BRADO1227	copper-containing nitrite reductase (NO-forming) nirK (EC:1.7.2.1)
gfo	GFO_1411	nitrous-oxide reductase (EC:1.7.2.4)
sme	SMa1182	nitrous-oxide reductase (EC:1.7.99.6)
bcs	BCAN_B0277	nitrous-oxide reductase
rge	RGE_19210	nitrous oxide reductase, NosZ (EC:1.7.2.4)
**Methane metabolism**		
rsc	RCFBP_11880	carbon monoxide dehydrogenase medium chain (EC:1.2.99.2)
rsc	RCFBP_11878	carbon monoxide dehydrogenase small chain (EC:1.2.99.2)
rme	Rmet_0363	putative carbon-monoxide dehydrogenase (acceptor) (EC:1.2.99.2)
rme	Rmet_0365	putative carbon monoxide dehydrogenase, 2Fe-2S iron-sulfur small subunit (EC:1.2.99.2)
smx	SM11_chr3168	putative carbon monoxide dehydrogenase medium subunit transmembrane protein
smx	SM11_chr3170	putative carbon monoxide dehydrogenase small subunit protein
ppf	Pput_0523	formate dehydrogenase subunit alpha
pmy	Pmen_0389	formate dehydrogenase subunit alpha

mca	MCA1210	formate dehydrogenase subunit alpha (EC:1.2.1.2)
she	Shewmr4_3250	hydrogenases, Fe-only (EC:1.12.7.2)
shn	Shewana3_0710	iron hydrogenase, small subunit
dds	Ddes_1502	ferredoxin hydrogenase (EC:1.12.7.2)
rsp	RSP_0492	hupU hydrogenase small subunit
sp|P31005|MEDH_BACMT		NAD-dependent methanol dehydrogenase
**Methanogenesis**		
mmh	Mmah_0679	methylenetetrahydromethanopterin dehydrogenase (EC:1.5.99.9)
mtp	Mthe_0809	F420-dependent methylenetetrahydromethanopterin dehydrogenase (EC:1.5.99.9)
mel	Metbo_0080	F420-dependent methylenetetrahydromethanopterin dehydrogenase (EC:1.5.99.9)
apo	Arcpr_1586	methylenetetrahydromethanopterin dehydrogenase (EC:1.5.99.9)
mmd	GYY_01920	F420-dependent methylenetetrahydromethanopterin dehydrogenase (EC:1.5.99.9)
mmz	MmarC7_1126	H(2)-dependent methylenetetrahydromethanopterin dehydrogenase (EC:1.12.98.2)
mmx	MmarC6_0825	H(2)-dependent methylenetetrahydromethanopterin dehydrogenase (EC:1.12.98.2)
mmq	MmarC5_1551	H(2)-dependent methylenetetrahydromethanopterin dehydrogenase (EC:1.12.98.2)
mfs	MFS40622_0426	methyl-coenzyme M reductase, beta subunit (EC:2.8.4.1)
mif	Metin_0285	methyl-coenzyme M reductase, beta subunit (EC:2.8.4.1)
mmq	MmarC5_0021	methyl-coenzyme M reductase, beta subunit (EC:2.8.4.1)
mzh	Mzhil_0853	methyl-coenzyme M reductase, beta subunit (EC:2.8.4.1)
mst	Msp_0318	MrtB (EC:2.8.4.1)
mep	MPQ_1570	formylmethanofuran dehydrogenase subunit a
mep	MPQ_1572	formylmethanofuran dehydrogenase subunit c
mep	MPQ_1569	formylmethanofuran dehydrogenase
say	TPY_3706	tungsten formylmethanofuran dehydrogenase subunit E
rba	RB9834	formylmethanofuran dehydrogenase subunit A (EC:1.2.99.5)
rba	RB9836	tungsten formylmethanofuran dehydrogenase, subunit C (FwdC)
mmp	MMP1248	tungsten containing formylmethanofuran dehydrogenase subunit A (EC:1.2.99.5)
mmp	MMP1691	tungsten containing formylmethanofuran dehydrogenase subunit B (EC:1.2.99.5)
mmp	MMP1244	tungsten containing formylmethanofuran dehydrogenase subunit H (EC:1.2.99.5)
mmp	MMP0508	molybdenum containing formylmethanofuran dehydrogenase subunit E (EC:1.2.99.5)
mac	MA0304	formylmethanofuran dehydrogenase subunit E
mac	MA0833	formylmethanofuran dehydrogenase subunit A
**Nitrogen Fixation**		
afr	AFE_1520	nitrogenase molybdenum-iron protein subunit beta (EC:1.18.6.1)
afr	AFE_1521	nitrogenase molybdenum-iron protein subunit alpha (EC:1.18.6.1)
afr	AFE_1522	nitrogenase reductase (EC:1.18.6.1)
bge	BC1002_6929	nitrogenase iron protein (EC:1.18.6.1)
bge	BC1002_6930	nitrogenase molybdenum-iron protein alpha chain
bge	BC1002_6931	nitrogenase molybdenum-iron protein beta chain (EC:1.18.6.1)
azl	AZL_007690	nitrogenase molybdenum-iron protein subunit beta
azl	AZL_007700	nitrogenase molybdenum-iron protein subunit alpha
azl	AZL_007710	nitrogenase iron protein
**Nitrogen Metabolism**		
xcp	XCR_0350	arginase
xac	XAC4009	arginase
smz	SMD_0276	Arginase (EC:3.5.3.1)
rsc	RCFBP_21298	arginase (EC:3.5.3.1)
thi	THI_2516	Arginase (EC:3.5.3.1)
ret	RHE_CH03851	arginase (EC:3.5.3.1)
oni	Osc7112_5685	assimilatory nitrate reductase (ferredoxin) precursor (EC:1.7.7.2)
mar	MAE_53960	ferredoxin-nitrate reductase
anb	ANA_C13775	nitrate reductase (EC:1.7.99.4)
sus	Acid_2928	assimilatory nitrate reductase (NADH) subunit alpha apoprotein (EC:1.7.1.1)
art	Arth_3951	nitrate reductase (NADH)
har	HEAR1188	SoxC protein (EC:1.7.1.1 1.8.3.1)
pae	PA3875	respiratory nitrate reductase subunit alpha
pae	PA3874	respiratory nitrate reductase subunit beta
pae	PA3872	respiratory nitrate reductase subunit gamma
par	Psyc_0605	respiratory nitrate reductase subunit alpha apoprotein (EC:1.7.99.4)
par	Psyc_0606	respiratory nitrate reductase subunit beta (EC:1.7.99.4)
par	Psyc_0608	respiratory nitrate reductase subunit gamma (EC:1.7.99.4)
sbm	Shew185_3711	cytochrome c552
pmp	Pmu_13000	formate-dependent cytochrome c nitrite reductase, c552 subunit (EC:1.7.2.2)
vex	VEA_003114	cytochrome c552 (EC:1.7.2.2)
csy	CENSYa_0360	ferredoxin-sulfite/nitrite reductase (EC:1.7.7.1)
mex	Mext_4492	ferredoxin-nitrite reductase
mno	Mnod_2867	ferredoxin-nitrite reductase
hel	HELO_3235	assimilatory nitrite reductase, NAD(P)H, large subunit (EC:1.7.1.4)
hel	HELO_3236	assimilatory nitrite reductase, NAD(P)H, small subunit (EC:1.7.1.4)
rme	Rmet_4818	assimilatory nitrite reductase (NAD(P)H-binding) large subunit (EC:1.7.1.4)
rme	Rmet_4819	nitrite reductase, NAD(P)H-binding, small subunit (EC:1.7.1.4)
**Reserve/Reductive TCA**		
cpb	Cphamn1_1351	ATP-citrate lyase/succinyl-CoA ligase
cpb	Cphamn1_1350	ATP-grasp domain-containing protein
saf	SULAZ_0527	ATP-citrate synthase (ATP-citrate (pro-S-)-lyase)(citrate cleavage enzyme) (EC:2.3.3.8)
saf	SULAZ_0528	ATP-citrate synthase (ATP-citrate (pro-S-)-lyase)(citrate cleavage enzyme) (EC:2.3.3.8)
pmx	PERMA_1714	ATP-citrate synthase (ATP-citrate (pro-S-)-lyase)(citrate cleavage enzyme) (EC:2.3.3.8)
pmx	PERMA_1715	ATP-citrate synthase (ATP-citrate (pro-S-)-lyase)(citrate cleavage enzyme) (EC:2.3.3.8)
hpp	HPP12_1074	pyruvate flavodoxin oxidoreductase subunit delta
hpp	HPP12_1075	pyruvate flavodoxin oxidoreductase subunit alpha
hpp	HPP12_1076	pyruvate ferredoxin oxidoreductase beta subunit
hpp	HPP12_1073	pyruvate flavodoxin oxidoreductase subunit gamma
ctc	CTC02525	pyruvate synthase subunit porB (EC:1.2.7.1)
ctc	CTC02526	pyruvate synthase subunit porA (EC:1.2.7.1)
ctc	CTC02527	pyruvate synthase subunit porD (EC:1.2.7.1)
ctc	CTC02528	pyruvate synthase subunit porC (EC:1.2.7.1)
sku	Sulku_1458	2-oxoglutarate ferredoxin oxidoreductase subunit delta (EC:1.2.7.3)
sku	Sulku_1455	2-oxoglutarate ferredoxin oxidoreductase subunit gamma (EC:1.2.7.3)
sku	Sulku_1045	pyruvate ferredoxin/flavodoxin oxidoreductase subunit beta (EC:1.2.7.3)
sku	Sulku_1044	pyruvate flavodoxin/ferredoxin oxidoreductase domain-containing protein
gi|46849514|dbj|BAD17844.1|		citryl-CoA synthetase large subunit
gi|46849517|dbj|BAD17846.1|		citryl-CoA synthetase small subunit
tr|Q75VW6|Q75VW6_HYDTH		citryl-CoA synthetase small subunit
tr|A1Y9I5|A1Y9I5_9AQUI		Citryl-CoA lyase
tr|A1Y9I6|A1Y9I6_9AQUI		Citryl-CoA lyase
tr|A1Y9I4|A1Y9I4_9AQUI		Citryl-CoA lyase
gi|38602676|dbj|BAD02487.1|		2-oxalosuccinate reductase
gi|116234990|dbj|BAF34931.1|		2-oxoglutarate carboxylase small subunit

gi|116234991|dbj|BAF34932.1|		2-oxoglutarate carboxylase large subunit
**Sulfur Metabolism**		
mxa	MXAN_2334	sulfite reductase subunit beta (EC:1.8.1.2)
mxa	MXAN_2335	sulfite reductase [NADPH flavoprotein, alpha-component (EC:1.8.1.2)
agr	AGROH133_03082	sulfite reductase [NADPH flavoprotein subunit alpha (EC:1.8.1.2)
agr	AGROH133_05967	sulfite reductase [NADPH hemoprotein subunit beta (EC:1.8.1.2)
rcp	RCAP_rcc01594	sulfite reductase (NADPH) hemoprotein subunit beta (EC:1.8.1.2)
rcp	RCAP_rcc03007	sulfite reductase (NADPH) flavoprotein subunit alpha (EC:1.8.1.2)
dgo	DGo_CA1712	Ferredoxin-nitrite reductase
rop	ROP_09710	sulfite reductase (EC:1.8.7.1)
mmar	MODMU_1123	sulfite reductase (EC:1.8.7.1)
tth	TTC1046	sulfite dehydrogenase soxC precursor (EC:1.8.2.1)
rde	RD1_1516	sulfite dehydrogenase SoxC protein (EC:1.8.2.1)
reh	H16_A3572	sulfite dehydrogenase (SoxC) (EC:1.8.2.1)
mtn	ERDMAN_1937	molybdopterin oxidoreductase
rha	RHA1_ro01563	sufite oxidase (EC:1.8.3.1)
hau	Haur_2864	sulfite oxidase
**Wood-Ljunghal**		
dev	DhcVS_604	carbon monoxide dehydrogenase subunit alpha
dak	DaAHT2_0825	CO dehydrogenase/acetyl-CoA synthase complex, beta subunit (EC:2.3.1.169)
dsf	UWK_03162	CO-methylating acetyl-CoA synthase precursor
dol	Dole_3184	bifunctional acetyl-CoA decarbonylase/synthase complex subunit alpha/beta (EC:2.3.1.169)
dal	Dalk_0681	bifunctional acetyl-CoA decarbonylase/synthase complex subunit alpha/beta (EC:2.3.1.169)
dmi	Desmer_2535	bifunctional acetyl-CoA decarbonylase/synthase complex subunit alpha/beta (EC:2.3.1.169)
adg	Adeg_0336	bifunctional acetyl-CoA decarbonylase/synthase complex subunit alpha/beta (EC:2.3.1.169)
**3-Hydroxypropionate Cycle**		
gi|119459821|gb|EAW40916.1|		short chain dehydrogenase
tr|A0Z522|A0Z522_9GAMM		short chain dehydrogenase
tr|A7NN59|A7NN59_ROSCS		short chain dehydrogenase
tr|W9BSS1|W9BSS1_9SPHN		short chain dehydrogenase
tr|A5UY60|A5UY60_ROSS1		Acrylyl-CoA reductase (NADPH) / 3-hydroxypropionyl-CoA dehydratase / 3-hydroxypropionyl-CoA synthetase
tr|A4A7V6|A4A7V6_9GAMM		3-hydroxypropionyl-CoA synthetase/3-hydroxypropionyl-CoA dehydratase/acrylyl-CoA reductase (NADPH)
tr|A9WBQ8|A9WBQ8_CHLAA		Acetyl-coenzyme A carboxylase carboxyl transferase subunit alpha
sp|A9WBQ9|ACCD_CHLAA		Acetyl-coenzyme A carboxylase carboxyl transferase subunit beta
tr|A9WBW5|A9WBW5_CHLAA		Acetyl-CoA carboxylase, biotin carboxyl carrier protein
tr|A9W9X0|A9W9X0_CHLAA		Acetyl-CoA carboxylase, biotin carboxyl carrier protein
tr|A9WIU3|A9WIU3_CHLAA		short chain dehydrogenase
tr|Q8VRG6|Q8VRG6_CHLAU		Propionyl-CoA synthase
tr|A9WKJ2|A9WKJ2_CHLAA		Propionyl-CoA carboxylase
tr|A9WEI4|A9WEI4_CHLAA		Propionyl-CoA carboxylase
tr|A9WGS0|A9WGS0_CHLAA		Glyoxalase/bleomycin resistance protein/dioxygenase
tr|A9WI24|A9WI24_CHLAA		Methylmalonyl-CoA mutase, large subunit
tr|A9WI23|A9WI23_CHLAA		Methylmalonyl-CoA mutase, large subunit
sp|A9WC39|SMTB_CHLAA		Succinyl-CoA--L-malate CoA-transferase beta subunit
sp|A9WC40|SMTA_CHLAA		Succinyl-CoA--L-malate CoA-transferase alpha subunit
sp|A9WC35|MCLA_CHLAA		Malyl-CoA/beta-methylmalyl-CoA/citramalyl-CoA lyase
**3-Hydroxypropionate/4-Hydroxybutyrate Cycle**		
640506050	Msed_0147	Acetyl-CoA carboxylase subunit A
640506051	Msed_0148	Acetyl-CoA carboxylase subunit B
640507264	Msed_1375	propionyl-CoA carboxylase carboxyltransferase subunit alpha / propionyl-CoA carboxylase carboxyltransferase subunit beta
640506613	Msed_0709	succinyl-CoA reductase (NADPH) (EC 1.2.1.-)
640507344	Msed_1456	3-Hydroxyproprionyl-CoA synthetase
640507889	Msed_2001	3-Hydroxypropionyl-CoA dehydratase
640507315	Msed_1426	Acryloyl-CoA reductase
640506543	Msed_0639	methylmalonyl-CoA epimerase (EC 5.1.99.1)
640506542	Msed_0638	methylmalonyl-CoA mutase (EC 5.4.99.2)
640507945	Msed_2055	cobalamin B12-binding domain protein
640507313	Msed_1424	succinate semialdehyde reductase (NADPH) (EC 1.1.1.-)
640507311	Msed_1422	4-hydroxybutyryl-CoA synthetase
640507112	Msed_1220	4-hydroxybutyryl-CoA dehydratase
640507210	Msed_1321	4-hydroxybutyryl-CoA dehydratase
640506305	Msed_0399	Crotonyl-CoA hydratase Candidate A/3-Hydroxybutyryl-CoA dehydrogenase Candidate B
640506290	Msed_0384	Crotonyl-CoA hydratase Candidate B
640506291	Msed_0385	Crotonyl-CoA hydratase Candidate C
640506242	Msed_0336	Crotonyl-CoA hydratase Candidate D
640506470	Msed_0389	Crotonyl-CoA hydratase Candidate E
640507312	Msed_1423	3-hydroxybutyryl-CoA dehydrogenase Candidate A
640507881	Msed_1993	3-hydroxybutyryl-CoA dehydrogenase Candidate C
640506295	Msed_0389	3-hydroxybutyryl-CoA dehydrogenase Candidate D
640506560	Msed_0656	Acetoacetyl-CoA beta-ketothiolase Candidate A
640507538	Msed_1647	Acetoacetyl-CoA beta-ketothiolase Candidate B
640507180	Msed_1290	Acetoacetyl-CoA beta-ketothiolase Candidate C
640506302	Msed_0396	Acetoacetyl-CoA beta-ketothiolase Candidate D
640506292	Msed_0386	Acetoacetyl-CoA beta-ketothiolase Candidate E
640506177	Msed_0271	Acetoacetyl-CoA beta-ketothiolase Candidate F
640506176	Msed_0270	Acetoacetyl-CoA beta-ketothiolase Candidate G

Table S3.

**Based on RAST annotations**	SPGG1	SPGG2	SPGG3	SPGG4	SPGG5	SPGG6	SPGG7	SPGG8	SPGG9
• Amino acid	1	1	-	-	-	-	-	-	2
• Antimicrobial peptide	-	-	-	-	-	-	-	-	1
• Arginine/ornithine	-	2	-	-	-	-	-	-	-
• Branched amino acid	16	-	-	-	1	-	-	-	2
• Dipeptide	4	6	1	3	-	2	4	-	6
• Drug/metabolite	1	3	1	4	2	1	-	3	1
• Glutamate/Aspartate	-	-	-	-	-	1	-	-	1
• Glycine	-	-	-	-	1	-	-	-	-
• H/L/R/O Amino acids	-	1	-	-	-	-	-	-	1
• High affinity branched amino acid	3	-	-	-	-	-	-	-	1
• Histidine	-	2	-	-	-	-	-	-	-
• L-proline/glycine	1	3	-	-	1	-	1	-	-
• Methione	1	-	-	-	-	1	1	1	-
• Oligopeptide	13	12	9	3	-	2	7	7	3
• Peptide	-	1	-	-	-	-	-	-	-
• Polar amino acid	1	3	1	-	-	-	-	-	-
• Taurine	-	1	-	-	-	-	-	-	-
Alpha glucoside	-	-	-	-	-	-	-	-	1
Ferric iron	12	5	-	1	1	3	2	-	-
Glycerol-3-phosphate	3	-	-	-	1	-	-	-	-
Lipopolysaccharide	2	1	1	-	-	1	2	1	-
Maltose/maltodextrin	-	-	-	-	-	2	-	-	1
Molybdenum	-	-	1	-	1	-	-	-	-
N-acetyl-D-glucosamine	-	-	-	-	1	-	-	-	-
Nucleoside	-	2	-	-	-	-	2	-	-
Organic solvent	4	1	1	1	-	-	1	-	-
Pd/Cd/Zn/Hg	-	-	-	-	-	1	-	-	-
Phosphate	-	2	4	-	1	-	1	-	-
Phosphonate	5	3	2	-	-	1	-	-	-
Putrescine/spermidine	-	2	-	-	3	1	2	-	-
Pyrimidine	2	-	-	-	-	-	1	-	-
Ribose	1	-	-	-	-	-	-	-	-
Sugar	1	-	-	-	1	-	-	-	1
Thiamin (B1)	1	-	-	-	-	-	3	-	-
Tungstate	3	4	-	-	-	-	3	-	-
Vitamin B12	-	2	1	1	-	3	-	-	-
Zinc	3	3	-	4	-	-	2	-	-
Unassigned	14	19	5	9	9	10	9	24	4
∑	92	79	27	26	23	29	41	36	25

Table S4.

	SPGG1	SPGG2	SPGG3	SPGG4	SPGG5	SPGG6	SPGG7	SPGG8	SPGG9	Unbinned Thaumarchaeota	Unbinned Gammaproteobacteria
**Phosphorous uptake**											
High affinity transporters (PstSABC)	-	-	+ (PstABC)	-	+ (PstS)	-	-	-	-	-	+ (PstBC)
Phosphonate transporters	+	+	+	-	-	-	-	+	-	+	+
Low affinity phosphate transporters	+	+	-	+	-	-	+	-	-	-	+
**Vitamin Biosynthesis/Transport**											
Thiamin (B1) synthesis	+	-	+	+	+	-	-	-	-	-	-
Thiamin (B1) transporters	+	-	-	-	-	+	+	-	-	-	+
Cobalamin (B12) biosynthesis	-	-	-	-	-	-	-	-	-	+	-
Cobalamin (B12) transporters	-	-	+	-	-	-	-	-	-	-	+
Pyridoxine (B6) synthesis	-	-	-	-	-	-	+	-	-	-	-

Data S1. Newick tree format for Figure 2. (Remove ‘newlines’ after copying to a plain text file)

```
(((((((group13Nitrospiraceae:0.21092964655971957466,CandidatusNitrospiradefluvii:
0.14543188160630807215)100:0.17721501832976638280,(LeptospirillumferriphilumYSK:
0.00155637295155256622,LeptospirillumferriphilumML04:0.00000100000050002909)100:0.44948632
801406285253)100:0.06806413256201616913,ThermodesulfovibrioyellowstoniiDSM11347:0.41082052
035498595188)97:0.06575032177384075582,((NitrospinaspAB629B18:0.47016802808952545822,
(NitrospinaspSCGCAAA288L16:0.50265668289676945779,group635Desulfobacterales:
0.11978114600871617168)95:0.13551098993090524014)94:0.25043569275565347176,
((((((TistrellamobilisKA081020065:0.19167756477611891386,((group774Rhodospirrilaceae:
0.19101364469964590698,group775Rhodospirrilaceae:
0.20017999696189919812)100:0.33573234955125003109,
(((NovosphingobiumaromaticivoransDSM12444:0.10617463023924440801,
(SphingopyxisalaskensisRB2256:0.08900201684788146272,Sphingobiumchlorophenolicum:
0.06641060417447908204)70:0.02444848936685201438)86:0.02355619017689073932,
(SphingomonaswittichiiRW1:0.07099233427014736697,ZymomonasmobilissubspmobilisATCC10988:0.1
1313811726781800027)100:0.03801132005539338554)100:0.23075238032192127435,
(((((HyphomicrobiumdenitrificansATCC51888chromosome:
0.02092151917730271718,Hyphomicrobiumdenitrificans1NES1:0.01042669370620657489)100:0.25228
742036293932971,RhodomicrobiumvannieliiATCC17100chromosome:
0.17955846202523495503)65:0.04046376387134472535,
(((RhizobiumleguminosarumbvtrifoliiWSM1325:0.05387512702187897268,SinorhizobiummedicaeWSM4
19:0.03470989666230372972)100:0.06236250822940978683,
(ChelativoransspBNC1:0.06522971548696819555,
((MesorhizobiumopportunistumWSM2075finalversionfromORNL:0.00535341108410514969,
(MesorhizobiumciceribiovarbiserrulaeWSM1271chromosome:
0.00804436325916575538,MesorhizobiumaustralicumWSM2073:0.00557532645923227398)85:0.0067000
1799815315904)100:0.07399059316895574556,
(OchrobactrumanthropiATCC49188chromosome1:0.01411975946857613358,BrucellacanisATCC23365chr
omosomeI:
0.01522995674657252885)100:0.08091974069904238342)72:0.02129471441471275650)87:0.024568407
52601791023)99:0.10822901226618332482,
(((RhodopseudomonaspalustrisTIE1:0.03711411885335493682,
((NitrobacterwinogradskyiNb255:0.01493440117869005053,NitrobacterhamburgensisX14:0.0222142
4483063152988)100:0.04349526098545804242,BradyrhizobiumspBTAi1:0.04389582680107195767)84:0
.01527428425620405185)100:0.10385253588341207542,StarkeyanovellaDSM506chromosome:
0.11291505064601567443)41:0.02344569403074218267,
((MethylocellasilvestrisBL2:0.05464043038036633937,BeijerinckiaindicasubspindicaATCC9039:0
.
07229095614446159890)100:0.07916111076151211012,Methylobacteriumsp446:0.115106430521686661
78)89:0.02800633294853319133)100:0.06842757361588072684)75:0.03231828339977459857)83:0.032
73520478094273101,(((AsticcacaulisexcentricusCB48chromosome1:0.07139716903431501949,
(BrevundimonassubvibrioidesATCC15264chromosome:0.10974382535984560094,
(CaulobacterspK31:0.02669868677649127331,
(AsticcacaulisexcentricusCB48chromosome2:0.22850902402884135101,CaulobactersegnisATCC21756
chromosome:
0.02801530432666107795)100:0.20786963386175413837)99:0.04819269328819669740)75:0.030271264
91166191175)99:0.15352979221452625613,
(MaricaulismarisMCS10:0.15613674800247712149,HirschiabalticaATCC49814:0.214338476651446396
83)100:0.05265417581917922890)98:0.04714702065892600186,
((RhodobactersphaeroidesATCC17025:0.07981608190509724421,ParacoccusdenitrificansPD1222chro
mosome1:0.09080646209551171411)100:0.04189173209496093592,
(JannaschiaspCCS1:0.07330175767273366949,
((SilicibacterspTM1040:0.00000100000050002909,RuegeriaspTM1040:0.00049573655088830566)100:
0.07990490437940972468,DinoroseobactershibaeDFL12:0.06450492356596485011)36:0.018415886837
39149579)100:0.05626746350971384425)100:0.21197308893209837466)36:0.02613930429306726005)7
3:0.03155959185738886619,
(ParvibaculumlavamentivoransDS1:0.12669881448843223204,CanditatusPhaeomarinobacterectocarp
iEc32:0.17619898815214019017)100:0.03904864356828247757)74:0.03205514085961270326)52:0.039
02226076201840282,(((MagnetospirillummagneticumAMB1:0.15125725695333089305,
((RhodospirillumrubrumF11:0.00049767018339412321,
(RhodospirillumrubrumATCC11170:0.00000100000050002909,RhodospirillumrubrumS1ATCC11170:0.00
049759289693341105)66:0.00000100000050002909)100:0.08224997648183392240,Rhodospirillumphot
```

ometricumDSM122:0.10144084531649466552)100:0.10557612483438801043)62:0.0298383697189394184
2,(RhodospirillumcentenumSW:0.09412844474209934587,
((AzospirillumspB510:0.02428457315376439385,Azospirillumlipoferum4B:
0.01693772020963617286)100:0.10252371169241233928,
(AzospirillumbrasilenseAz39:0.00341674795530730584,AzospirillumbrasilenseSp245:0.004157089
03083724258)100:0.04170989393970397224)100:0.07094345514655828189)100:0.092651320106361692
57)15:0.01746341423304809473,
((((ThalassospiraxiamenensisM5DSM17429:0.00071161354583189546,ThalassospirapermensisNBRC10
6175:0.00391962281813046723)100:0.02032033237366959177,ThalassospiralucentensisDSM14000:0.
03359710307099804394)100:0.18214348121447693218,TerasakiellapusillaDSM6293:0.2102514086263
4306149)74:0.03916937612431539012,(AcidiphiliumcryptumJF5:0.39451393371040271996,
(CandidatusEndolissoclinumfaulkneriL5:0.04177157656059198032,CandidatusEndolissoclinumpate
llaL2:0.04391543505998502656)100:0.25032236278275787900)26:0.03672570966281758081)8:0.0110
2626593691682054)63:0.02920161776630335326)15:0.01908024155258597823)10:0.0114330447738091
4681)65:0.06414770613514933495,EhrlichiacanisJake:
0.68956763084676575559)71:0.14427435387694401148,MagnetococcusspMC1:0.32577332185084256766
)62:0.05558750383834375891,
(((zetaproteobacteriumSCGCAB604B04:0.01540629012476852494,zetaproteobacteriumSCGCAC673M07:
0.01239510754091588916)100:0.17316673983275435167,
((zetaproteobacteriumSCGCAB137L23:0.00000100000050002909,zetaproteobacteriumSCGCAB137C09:0
.00000100000050002909)100:0.17473983344073246160,
((ZetaproteobacteriabacteriumTAG1:0.17751246608947415795,
(zetaproteobacteriumSCGCAB133C04:0.01644641683475883687,zetaproteobacteriumSCGCAB137I08:0.
05232664313852970761)100:0.12228855481667021132)72:0.03948946413545192935,
(MariprofundusspEKFM39:0.07095267467231901326,
(MariprofundusferrooxydansPV1unfinishedsequence:
0.00247242855668474478,MariprofundusferrooxydansM34:0.00111327833843855874)100:0.084996179
50578490511)100:0.06285334451394775090)43:0.02628560745955518460)100:0.0822836374929839797
8)100:0.24177490997498116143,(group636Gammaproteobacteria:1.12855161267359016541,
((AcidithiobacillusferrooxidansATCC53993:0.02613611769427010359,Acidithiobacillusferrivora
nsSS3:0.02580646096240065898)100:0.33026311023456650373,
(((NitrosococcushalophilusNc4chromosome:0.03868102273905160010,
(NitrosococcuswatsoniC113chromosome:
0.02098375553976130833,NitrosococcusoceaniATCC19707:0.01711130554061455339)100:0.062610018
56456141490)100:0.18747506008312067594,
(((((AcinetobactertjernbergiaeDSM14971:0.13128866871528876992,
(PsychrobactercryohalolentisK5:0.06407113710234568771,PsychrobacterspPRwf1:0.0616451074962
6712915)100:0.13713863794958280895)100:0.16706949309126595260,
((Saccharophagusdegradans240:0.16953266870721636628,(AzotobactervinelandiiDJ:
0.03351846267897069104,(PseudomonasstutzeriRCH2:0.04555010480571992232,
(Pseudomonasmendocinaymp:0.03018409095773354200,Pseudomonasfulva12X:
0.02305900623483517903)97:0.02135383020228004067)99:0.02438519373152439607)100:0.132329725
77088517041)35:0.03032347022008351906,(MarinobacteraquaeoleiVT8:0.15389711611103421007,
((OceanobacterkriegiiDSM6294:0.16384184467389037443,
(MarinomonasmediterraneaMMB1:0.03239317151988087978,
(MarinomonasspMWYL1:0.04090347852184276350,MarinomonasposidonicaIVIAPo181:0.01633155413342
024867)100:0.02322198446377430128)100:0.13883303783661851694)67:0.03247045867822848098,Chr
omohalobactersalexigensDSM3043:0.17907246729611892011)56:0.02524407729491529376)26:0.01817
044720418348192)98:0.04539567585571804148)73:0.02987071825642301920,
(KangiellakoreensisDSM16069:0.18484439760501847339,
(((RheinheimerabalticaDSM14885:0.01663111703914958023,RheinheimerapacificaDSM17616:0.01820
946389023375342)100:0.03636691763457505777,RheinheimeratexasensisDSM17496:0.04540459132286
976673)100:0.10874274246866312554,(((PseudoalteromonasatlanticaT6c:
0.00723918802080836688,Glaciecolasp4H37YE5chromosome:
0.00853614990873605324)100:0.13971644689640846937,
(Psychromonasingrahamii37:0.19870692957205657314,(FerrimonasbalearicaDSM9799chromosome:
0.10426054445595536735,
(ShewanellabalticaBA175:0.03239409830203310997,ShewanelladenitrificansOS217:0.039997720831
24075728)100:0.10307175750011753068)88:0.03162806216906433043)96:0.02784209390032475750)99
:0.02926063915710078525,(TolumonasauensisDSM9187:0.12430470750184477446,
(((YersiniapestisNepal516:0.04038516550426621970,
(((SerratiaspAS13:0.00599585536765616118,Serratiaproteamaculans568:0.00757351487301267251)
100:0.00971080557279372508,
(RahnellaaquatilisCUETM77115:0.00092330594711480989,RahnellaspY9602chromosome:

0.00157284619960445572)100:0.02212863206825911241)100:0.01669311829468875588,
(((KlebsiellavariicolaAt22chromosome:0.01209401912121182109,
(EnterobactercloacaeSCF1chromosome:0.00696844369818861060,
(Enterobactersp638:0.03218145515283939162,
(CronobacterspFGI57:0.00944081186204049315,EscherichiacoliATCC8739:0.01644092872621664633)
66:0.00537366002784594229)57:0.00411576503182118844)71:0.00876616622588446796)98:0.0152294
1668645196207,(PantoeaspAt9bchromosome:0.03109868082431810343,
((DickeyadadantiiEch703:0.01275214688420849181,
(DickeyadadantiiEch586:0.00493546686208044642,DickeyazeaeEch1591:0.00722074151898815034)90
:0.00710405167515427809)100:0.02085233072160252998,
(PectobacteriumcarotovorumsubspcarotovorumPC1:0.00376376486994457532,Pectobacteriumwasabia
eWPP163:0.00528266094057915688)100:0.02512703892348513990)85:0.01380902395296622689)20:0.0
0157571408860370768)66:0.01684709050640758113,SerratiaspFGI94:0.02089239415287688639)85:0.
01741542407718827887)86:0.02101741302842404652)99:0.02902672201301120802,Buchneraaphidicol
astrTuc7Acyrthosiphonpisum:0.26168589847126405568)99:0.03543180210364560434,
((Haemophilussomnus2336:0.00236459692081654634,(Haemophilussomnus129PT:
0.00000100000050002909,Histophilussomni129P:
0.00050064981455379121)98:0.00014531796373834285)100:0.06563136617968008679,Actinobacillus
succinogenes130Z:
0.04467349555806689188)100:0.09684968580695145490)100:0.05556345874874868418)100:0.0588791
1888557184614)52:0.01601277853960342279)100:0.08024099274451299002)97:0.043658261496497603
74)83:0.03725760601267735089,
(FrancisellatularensissubsptularensisWY963418:0.41657742252643209513,
(CandidatusRuthiamagnificastrCmCalyptogenamagnifica:0.44143730128704516957,
(ThiomicrospiracrunogenaXCL2:0.13679923999565232751,
(ThioalkalimicrobiumcyclicumALM1:0.02128908478353002537,ThioalkalimicrobiumaerophilumAL3:0
.
01467822113513179115)100:0.10820878436264240452)100:0.11616614730612505324)50:0.0451341541
8067122376)69:0.04403131332778828511)83:0.03171182516130826440,
((((AlkalilimnicolaehrlichiiMLHE1:0.08791440421811266837,HalorhodospirahalophilaSL1:0.1688
1229494695842996)98:0.04698554465897036847,
(ArhodomonasaquaeoleiDSM8974:0.07974984471631650640,
(EctothiorhodospiraceaebacteriumM1940:0.05997618806750611153,SpiribacterspUAHSP71:0.069015
02279098503667)100:0.09632021314540747947)100:0.07400816499284419703)100:0.111179353223831
73946,((HalorhodospirahalochlorisA:0.11614376301534391767,
(ThioalkalivibriospHLEbGR7:0.09020540651020907130,
((ThioalkalivibriospALJ24:0.08657355947294062548,(ThioalkalivibriospK90mixchromosome:
0.00483594417984504164,ThioalkalivibriospALJ2:0.00431830979739664979)100:0.042629738308181
47375)100:0.07208234500239088516,
(ThioalkalivibriothiocyanoxidansARh4:0.01376895000237900180,Thioalkalivibrionitratireducen
sDSM14787:0.02107939841838884013)100:0.05525004715144886391)100:0.11372734398823293456)92:
0.04145441842967499679)98:0.06891462551118275603,
(MethylomonasmecanicaMC09:0.27650541670822309515,
(Thioflavicoccusmobilis8321:0.11532506222614818947,
(Marichromatiumpurpuratum987:0.06383847123627618692,
(Thiocystisviolascens611DSM198:0.08309293665614468638,AllochromatiumvinosumDSM180chromosom
e:
0.06048855713228023373)71:0.02038577019868338669)100:0.04606061983883834310)100:0.10569810
712547758613)69:0.03553631917279778674)18:0.01292980715257867341)26:0.02500001126362528039
,(Halothiobacillusneapolitanusc2:0.28911137576269724248,
((((XylellafastidiosaM12:0.00271787874610259980,XylellafastidiosaM23:0.0041979925026944774
1)100:0.12827709820982594047,
(StenotrophomonasmaltophiliaR5513:0.10246757914436611936,Pseudoxanthomonassuwonensis111chr
omosome:0.06074092708381872291)84:0.02518768227957546985)100:0.07524660025418679343,
(Rhodanobactersp2APBS1:0.04131400816535611697,FrateuriaaurantiaKondo67DSM6220:0.1632549190
5603353726)100:0.08688762996347290657)100:0.16031940384072770889,
((HydrocarboniphagadaqingensisCGMCC17049:0.04839721743465211223,HydrocarboniphagaeffusaAP1
03:0.04465399476241448118)100:0.12515998073824613490,
(PolycyclovoransalgicolaTG408:0.15575397012985917056,
(SolimonasaquaticaDSM25927:0.04761792510710995130,
(SinobacterflavusDSM18980:0.00860969287110833169,SingularimonasvariicolorisDSM15731:0.0111
1823771469622885)100:0.03520859628352830673)100:0.10548416974621432907)76:0.03884011785653
748716)100:0.17301335731536943152)72:0.04306886582254623730)34:0.03242494698460621216)18:0
.

01609872916938879031)27:0.0266961910136916565l)83:0.03761850362388813318,group638Gammaprot
eobacteria:
0.28906620037089053588)85:0.04693307485478614982)83:0.05292982807350679608)57:0.0823334194
1642843831)52:0.04613403178923230347)91:0.12737363657243577775,
(((HaliangiumochraceumDSM14365:0.56123370159488128461,
(AnaeromyxobacterspFw1095:0.05601895713170434998,(Anaeromyxobacterdehalogenans2CPC:
0.00762014661025801811,
(Anaeromyxobacterdehalogenans2CP1:0.00201011351247502639,AnaeromyxobacterspK:
0.00050638126195885627)100:0.00152478591945618422)100:0.04394053002838000516)100:0.2469581
4655423992123)96:0.07747407154027478871,(((GeobacterlovleyiSZ:
0.08111257656826026397,PelobacterpropionicusDSM2379:0.08464546453572840967)100:0.054012810
37046137129,(((GeobacterspM18chromosome:0.01636000702565982429,
(GeobacterspM21:0.00582928118440401849,GeobacterbemidjiensisBem:
0.01170924622610534580)100:0.01949373299406139196)100:0.08250502187230134010,
(GeobacterspFRC32:0.02356448504981380199,GeobacteruraniumreducensRf4:0.0283783648951684355
8)100:0.03524766771563059981)100:0.04028074043370279134,GeobactermetallireducensGS15:0.093
68022419774268972)90:0.02189958685155818374)100:0.08651719000014698735,Pelobactercarbinoli
cusDSM2380:0.20998598136472551579)100:0.10560027509825786751)90:0.04345357770606432524,
((((SyntrophobacterfumaroxidansMPOB:0.33869229272885537085,
((DesulfococcusoleovoransHxd3:0.29997349809296819378,
(DesulfobaculatoluolicaTol2:0.19149575910271249279,DesulfobacteriumautotrophicumHRM2:0.138
63865378839801412)100:0.15528190761887267213)100:0.07229466312557124297,Desulfatibacilluma
lkenivoransAK01DesulfatibacillumalkenivoransAK01:0.26083952029076545864)100:0.156750283068
44551729)100:0.11418536600546491666,(DesulfarculusbaarsiiDSM2075chromosome:
0.33537095623710616588,DesulfobaccaacetoxidansDSM11109:0.28647623185125858924)99:0.0596513
1969245880683)91:0.03005789210583880225,
(((((DesulfovibriodesulfuricanssubspdesulfuricansstrATCC27774:0.14371390527266852954,
(DesulfovibriovulgarisstrMiyazakiF:
0.07136903072811384696,DesulfovibriovulgarissubspvulgarisDP4:0.07961269455186266442)88:0.0
3093195894936387816)100:0.04792729258069469006,
(DesulfovibriodesulfuricansG20:0.00000100000050002909,DesulfovibrioalaskensisG20:0.0005017
7715423409448)100:0.11516647899519305565)100:0.09024920869433418480,
(DesulfovibrioafricanusWalvisBayOctober2010draft:0.17312939545238906214,
((DesulfovibrioaespoeensisAspo2chromosome:
0.07331041143612021527,DesulfovibriodesulfuricansND132FinalJGIassembly:
0.06823917977362438381)100:0.13088145352706723190,DesulfovibriosalexigensDSM2638:0.1395463
1455043015054)100:0.07141613919822822898)84:0.03550702637290999858)99:0.048291563621199969
09,
(DesulfomicrobiumbaculatumDSM4028:0.23255762514366226656,DesulfohalobiumretbaenseDSM5692:0
.23670513031653003799)96:0.05360989128242953095)100:0.27254672951484376631,
(((DesulfobulbuspropionicusDSM2032chromosome:0.22690806583026101872,
(DesulfotaleapsychrophilaLSv54:0.19463964906792952103,DesulfocapsasulfexigensDSM10523:0.17
154964337565642429)100:0.08757318007574711738)100:0.10849265012477724013,Desulfurivibrioal
kaliphilusAHT2chromosome:0.27944956397295728223)100:0.24423553000806977331,
(ThermodesulfatatorindicusCIR29812DSM15286:0.14643678253744846218,
(ThermodesulfobacteriumspOPB45:0.08981285303159491917,ThermodesulfobacteriumcommuneDSM2178
:
0.09034708452567179193)100:0.26404442053904225718)100:0.21439718941317223444)98:0.06347846
536393739192)98:0.04823364028786282509)93:0.03654058338054278626,DesulfomoniletiedjeiDCB1D
SM6799:0.42205151749554536966)100:0.03970781822401360517)77:0.03128648454620189256)89:0.03
918623907381078852,(candidatedivisionKSB3bacteriumUASB270Ga0063343:0.41335168350399043025,
(CandidatusMethylomirabilisoxyferaspAustralia:0.19011602219596832053,
(CandidatedivisionNC10bacteriumCSP15XU15C:0.05871220206273290027,group16Unclassified:
0.09140158758372270154)100:0.13783095545792453662)100:0.21203101016406195045)89:0.06078084
072425003065)44:0.02085797876097689879)65:0.02449209905146989413)78:0.04846773061552926698
,
(((HippeamaritimaMH2DSM10411:0.25070671863689869552,DesulfurellaacetivoransA63DSM5264:0.35
507802567661522763)100:0.30127320404463969750,
((NitratifractorsalsuginisDSM16511chromosome:0.19510530643486201363,
(SulfuricurvumkujienseDSM16994chromosome:0.13510126760199522855,
(SulfurimonasautotrophicaDSM16294chromosome:
0.08497549918228211596,SulfurimonasdenitrificansDSM1251:0.09342460749315584234)100:0.06553
844513148247841)100:0.12740240678639258576)57:0.02877125625210516513,
((SulfurospirillumdeleyianumDSM6946:0.01793860012976182874,SulfurospirillumbarnesiiSES3:0.

02416271881625712062)100:0.16823312733622408643,ArcobacternitrofigilisDSM7299chromosome:
0.27060691032156747315)65:0.02676132341172727733)100:0.46125941248778229431)100:0.09043153
419397656489,(MucispirillumschaedleriASF457:0.24931704070518018179,
(DenitrovibrioacetiphilusDSM12809chromosome:0.22487440738763819770,
(FlexistipessinusarabiciMAS10DSM4947:0.21426623374620257234,
(CalditerrivibrionitroreducensDSM19672chromosome:
0.20211234062939953393,DeferribacterdesulfuricansSSM1:0.16084731580107691773)41:0.02562901
767687776958)97:0.03692232504182574315)87:0.04161592446657807337)100:0.2602339678951973711
6)82:0.05189336881727731055)72:0.03573371323984889797,
((ElusimicrobiumminutumPei191:0.49349856204132314108,CandidatusEndomicrobiumtrichonymphaes
ubmission450:0.46379406100724784867)100:0.20089630355966459074,candidatedivisionNKB19bacte
riumJGI0000077O16TAbiofilm001237:0.68673356037173138855)96:0.11195433322405930199)36:0.000
00100000050002909,(((((SAR406clusterbacteriumJGI0000113G11:0.02887452634109431704,
(SAR406clusterbacteriumSCGCAAA160I06:0.00553784149048956037,SAR406clusterbacteriumSCGCAAA2
98D23:0.16217926114317252062)62:0.04450697758583356534)100:0.37823314804613555884,SAR406cl
usterbacteriumSCGCAB629J13:0.33125063332020571183)100:0.24618935948364967237,
(CaldithrixabyssiLF13DSM13497:0.30438491397553180118,
((CandidatusSulciamuelleriDMINchromosome:
0.76711984875773087111,SpirosomalingualeDSM74:0.32529327790238582452)100:0.274252669794365
13370,(IgnavibacteriumalbumMat916JCM16511:0.12368962345896433730,MelioribacterroseusP3M:
0.15805703386665093113)100:0.20229875330723634530)68:0.05442721084097165707)30:0.043889873
11493407206)52:0.05671190931129804769,group23Unclassified:
0.70898976872745733679)95:0.14400322122000264180,
(((((candidatedivisionSR1bacteriumtaxon345lOR1recodedII:
1.03780188025745578884,candidatedivisionGN02bacteriumJGI0000069K10CrabSpa001277:0.99436440
126764802372)84:0.16589562612484642923,
(candidatedivisionOD1bacteriumSCGCAAA036E14CrabSpa001178:0.67476067415131779637,
(candidatedivisionOD1bacteriumSCGCAAA011A09DUSEL001189:0.44278762633710233576,
(candidatedivisionZB2bacteriumSCGCAAA255P19SAK001138:0.47087730590581228229,candidatedivis
ionOP11bacteriumSCGCAAA010E09DUSEL001100:0.32068615655992865898)55:0.06298363851109832490)
100:0.15367185631078178476)99:0.20198348785678393869)87:0.10077117433084298304,
(candidatedivisionOP11bacteriumSCGCAAA011L6Dusel001266:0.35564387953886505978,
(candidatedivisionOP11bacteriumSCGCAAA011B20DUSEL001197:0.38328724158622640150,candidatedi
visionOP11bacteriumSCGCAAA040P11CrabSpa001265:0.65330727016840517418)91:0.121262362706810
8614)100:0.37459908136579406213)89:0.10691617421747300065,
((CandidatusCaldatribacteriumsaccharofermentansOP977CS:
0.05387085532210064698,CandidatusCaldatribacteriumcaliforniense0P9cSCG:
0.18070402457584425204)100:0.41991782045599829187,
(CaldisericumexileAZM16c01NBRC104410:0.98996019375905341953,ThermodesulfobiumnarugenseNa82
DSM14796:0.61733419726051397269)85:0.10577856206212797596)44:0.06696274687425640693)20:0.0
4846730106589458575,((AcetohalobiumarabaticumDSM5501chromosome:0.43261484046210013643,
(((SelenomonassputigenaDSM20758:0.18750747947526991144,AcidaminococcusfermentansDSM20731:0
.28866516457498825332)100:0.14827452587483602731,
(CaldicellulosiruptorsaccharolyticusDSM8903:0.01964161138836281392,
(CaldicellulosiruptorobsidiansisOB47chromosome:0.00958868261029449491,
((Caldicellulosiruptorkristjanssonii177R1Bchromosome:
0.00637079067728334083,Caldicellulosiruptorhydrothermalis108chromosome:
0.00270953290513896569)38:0.00135815267274581029,
(AnaerocellumthermophilumDSM6725:0.00163396537447864343,Caldicellulosiruptorkronotskyensis
2002chromosome:
0.00288588553560893907)95:0.00349213671972814672)55:0.00303005961923230030)99:0.0204863067
0856271471)100:0.30876258301036590970)64:0.04456613082841430484,
((AcidimicrobiumferrooxidansDSM10331:0.70424618963110741099,ThermaerobactermarianensisDSM1
2885chromosome:
0.25227665100402668630)42:0.05489393561531688559,SyntrophothermuslipocalidusDSM12680chromo
some:
0.41806053534160975138)33:0.04440205062031757355)25:0.03167466429509727122)36:0.0332952234
2853392242,FimbriimonasginsengisoliGsoil348:0.83417475682014186056)34:0.043251154108896343
32)41:0.04475126808767695280)24:0.02884945007436430509):
3.34030467712352718834,NitrosopumilusmaritimusSCM1:3.34030467712352718834);
